# Medical LLMs: Fine-Tuning vs. Retrieval-Augmented Generation

**DOI:** 10.3390/bioengineering12070687

**Published:** 2025-06-24

**Authors:** Bhagyajit Pingua, Adyakanta Sahoo, Meenakshi Kandpal, Deepak Murmu, Jyotirmayee Rautaray, Rabindra Kumar Barik, Manob Jyoti Saikia

**Affiliations:** 1 Biomedical Sensors & Systems Lab, University of Memphis, Memphis, TN 38152, USA; 2 Department of Computer Science and Engineering, Odisha University of Technology and Research, Bhubaneswar 751003, India; 3 Department of Information Technology, Veer Surendra Sai University of Technology, Sambalpur 768018, India; 4 School of Computer Applications, KIIT Deemed to be University, Bhubaneswar 751003, India; 5 Electrical and Computer Engineering Department, University of Memphis, Memphis, TN 38152, USA

**Keywords:** large language models, healthcare, medical, retrieval-augmented generation, fine-tuning

## Abstract

Large language models (LLMs) are trained on huge datasets, which allow them to answer questions from various domains. However, their expertise is confined to the data that they were trained on. In order to specialize LLMs in niche domains like healthcare, various training methods can be employed. Two of these commonly known approaches are retrieval-augmented Generation and model fine-tuning. Five models—Llama-3.1-8B, Gemma-2-9B, Mistral-7B-Instruct, Qwen2.5-7B, and Phi-3.5-Mini-Instruct—were fine-tuned on healthcare data. These models were trained using three distinct approaches: retrieval-augmented generation (RAG) alone, fine-tuning (FT) alone, and a combination of both (FT+RAG) on the MedQuAD dataset, which covers a wide range of medical topics including disease symptoms, treatments, medications, and more. Our findings revealed that RAG and FT+RAG consistently outperformed FT alone across most models, particularly LLAMA and PHI. LLAMA and PHI excelled across multiple metrics, with LLAMA showing superior overall performance and PHI demonstrating strong RAG/FT+RAG capabilities. QWEN lagged behind in most metrics, while GEMMA and MISTRAL showed mixed results.

## 1. Introduction

The sudden acceleration of LLMs has greatly influenced natural language processing (NLP) and has allowed their deployment across numerous sectors [1], even healthcare. LLMs could revolutionize the practice of medicine by increasing the availability of data, helping to make diagnoses, and aiding in clinical decision-making. Such models are used increasingly to evaluate symptoms, detect disease, and return potential diagnoses, depending on the question posed. But the specialized nature of medical information requires ensuring that their answers are accurate, reliable, and contextually relevant. To address these issues, LLMs have to be fine-tuned with domain-specific information to respond efficiently to medical questions, especially on symptoms, disorders, and diseases.

This study aims to tailor a large language model (LLM) for medical use, with the objective of improving its capability to respond to questions about disease symptoms and medical disorders. Five pre-trained language models—Llama-3.1-8B [2], Gemma-2-9B [3], Mistral-7B-Instruct [4], Qwen2.5-7B [5], and Phi-3.5-Mini-Instruct [6]—were fine-tuned on domain-specific healthcare data to improve their performance in medical question answering tasks. The efficiency of the model was gauged from its ability to accurately interpret and answer medical questions, providing safe information on conditions and possible treatment. To adapt LLMs for the medical domain, we explored three key techniques: (1) standard fine-tuning [7,8], which updates the model’s internal parameters using domain-specific data; (2) retrieval-augmented generation (RAG) [9], which improves performance by leveraging external knowledge at inference time without modifying model weights; and (3) a hybrid FT+RAG approach, which applies RAG on top of a fine-tuned model.

Retrieval-augmented generation (RAG) is a technique that combines retrieval-based systems with generative functionalities, offering better capacity for the model to deliver accurate and context-specific responses. Through the incorporation of external knowledge retrieval systems, RAG allows LLMs to gain access to current medical information from trusted sources and incorporate it into their generation. This provides more accurate and informed responses to medical questions. Conversely, standard fine-tuning encompasses training the model on a selected domain-specific data set directly and fine-tuning its internal representation to enhance its understanding of medical concepts such as symptoms and diseases.

The focus of this work is to assess the efficacy of RAG and standard fine-tuning on medical query answering. This study assessed how each method improved the accuracy, relevance, and completeness of model answers and decided which technique is more appropriate for certain categories of medical questions. It also considered the trade-offs between these methods in terms of computation, data needs, and being able to utilize contemporary medical knowledge.

Through this comparative study, our research aims to offer useful insights into the creation of AI-based healthcare tools that can assist medical professionals and improve patient care. For large language models (LLMs), the paper [10] outlines the trade-offs between RAG and fine-tuning, pointing out the cost-effectiveness and contextual relevance of RAG and the higher accuracy of fine-tuning with large datasets. RAG has been demonstrated to provide a more stable and dependable method of incorporating external knowledge, even though fine-tuning is still effective in a variety of applications [11]. With the growing adoption of AI-powered healthcare solutions, it is crucial that language models employed in healthcare applications are both extremely accurate and contextually sensitive. This work will help to advance fine-tuning and adaptation techniques for LLMs in healthcare settings, enabling the creation of more effective, efficient, and trustworthy AI systems for healthcare.

### 1.1. Objective

This research aims to fine-tune five different LLMs for medical applications, with a focus on enhancing their ability to accurately respond to queries related to symptoms and disorders. Three methods—RAG, conventional fine-tuning, and a mix of both—are compared in this work to evaluate their efficacy in producing accurate, relevant, and context-aware responses. By assessing the performance of these approaches, our study aims to identify the most effective way to maximize LLMs in the healthcare sector, thus helping to produce more trustworthy artificial intelligence-driven tools for medical query answering.

### 1.2. Contributions

The researchers in this work trained and tested five 4-bit quantized large language models—Meta-Llama-3.1-8B, Phi-3.5-mini-instruct, Gemma-2-9B, Mistral-7B-instruct, and Qwen2.5-7B—on the Unsloth framework to support efficient fine-tuning. The main contributions of this work are as follows:

- It measures the impact on several models of three adaptation strategies: fine-tuning (FT), retrieval-augmented generation (RAG), and a hybrid FT+RAG approach.
- It addresses four central research questions: (1) Which model demonstrates the best overall performance? (2) Which adaptation method—fine-tuning, RAG, or FT+RAG—is the most effective overall? (3) Which model performs optimally with each specific adaptation method? (4) Which adaptation method yields the best results for each model?
- It applies a comprehensive evaluation framework employing both lexical and semantic metrics. The metrics used include BLEU, GLEU, ROUGE, METEOR, Precision, Recall, F1, BERTScore, SBERT cosine similarity, and Negation-Aware Semantic Similarity.
- Finally, it offers an overall perspective of model performance across many adaptation approaches.

### 1.3. Organizations

The organization of this study is structured as follows: Section 2 summarizes current studies on retrieval-augmented generation and fine-tuning for LLMs. Section 3 outlines our approach to evaluating five quantized LLMs across three adaptation methods—FT, RAG, and FT+RAG. The major components—datasets, LLMs, ChromaDB, and system configuration—are briefly discussed in Section 4. The preprocessing of the dataset, fine-tuning and RAG implementation strategies, and the evaluation plan are covered in Section 5. We present and analyze in Section 6 results including model training outcomes, perplexity comparisons, performance on lexical and semantic measures, key findings, and a performance comparison with other models. Section 7 lists current constraints as well as possible areas for development. Finally, Section 8 summarizes our main results and acquired knowledge on model–method performance alignment for tasks particular to a domain.

## 2. Related Work

This section discusses the related works we considered before performing our research, giving an overview of prior research on fine-tuning, retrieval-augmented generation, and their applications in domain-specific task optimization of LLMs.

Ref. [11] compared RAG and FT for injecting known and new knowledge into LLMs. They found that RAG outranks FT by a wide margin consistently, especially when models need to learn novel information outside of their training data.

Ref. [12] investigated the effect of RAG and fine-tuning for low-frequency domain knowledge in LLMs. They observed that although FT considerably improves performance for both frequent and infrequent entities, eventually, RAG delivers better accuracy, particularly when augmented using strong retrieval and augmentation practices.

Ref. [13] explored DSL code generation with fine-tuned Codex and optimized RAG. FT produced higher code similarity, but RAG was more flexible to unseen APIs and had a higher compilation rate, which indicates its value in fast-changing environments.

Ref. [14] utilized FT and RAG on medical chatbots with models such as LLaMA and Flan-T5. The research showed that the use of FT along with RAG enhanced QA accuracy under computational limits, especially for medical datasets.

Ref. [15] explored LLM adaptation for Portuguese QA. Their results showed that while both FT and RAG each contribute, a hybrid combination of FT+RAG provided the best domain adaptation and answer quality.

Ref. [16] benchmarked RAG against FT with GPT-J and LLaMA models for knowledge-based systems. RAG performed better than FT on metrics such as ROUGE and BLEU, although FT produced marginally higher creativity, implying use case priority-based trade-offs.

Ref. [17] compared the system-level trade-offs of RAG, commenting on its capability to skip retraining with the expense of added latency and memory requirements. They presented a taxonomy to balance performance and efficiency in deploying RAG pipelines.

Ref. [18] set up performance baselines with GPT-3.5 using FT, RAG, and soft-prompting. RAG had the highest accuracy on questions of post-2021 knowledge, with soft prompting adding to all setups.

Ref. [19] developed a preoperative medicine QA system with LLM-RAG pipelines. GPT-4 with RAG had 91.4% accuracy—higher than human physicians—showing the scalability, low hallucination rate, and good adherence to clinical guidelines of RAG.

Ref. [20] provided a survey that classified the types and challenges of RAG tasks. They proposed frameworks that combined external data through context, small models, or fine-tuning, with a focus on the necessity of hybrid approaches in real-world, complex LLM applications.

Table 1 is a compilation of all the literature on fine-tuning vs. RAG of LLMs that we reviewed. These studies were important to understand the earlier work on fine-tuning and RAG in LLMs as well as what kind of datasets are used generally. They offered useful insights that informed the design and direction of our work.

## 3. Proposed Framework

Three methodological approaches—fine-tuning, retrieval-augmented generation (RAG), and their hybrid combination—were implemented to enhance the models’ medical QA capabilities. Training the models on healthcare-specific datasets helps to create specialized versions fit for medical searches by means of fine-tuning. RAG improved response quality instead by including external knowledge retrieval systems. Combining the advantages of fine-tuning with RAG was the hybrid approach meant to maximize performance.

**Figure 1:**
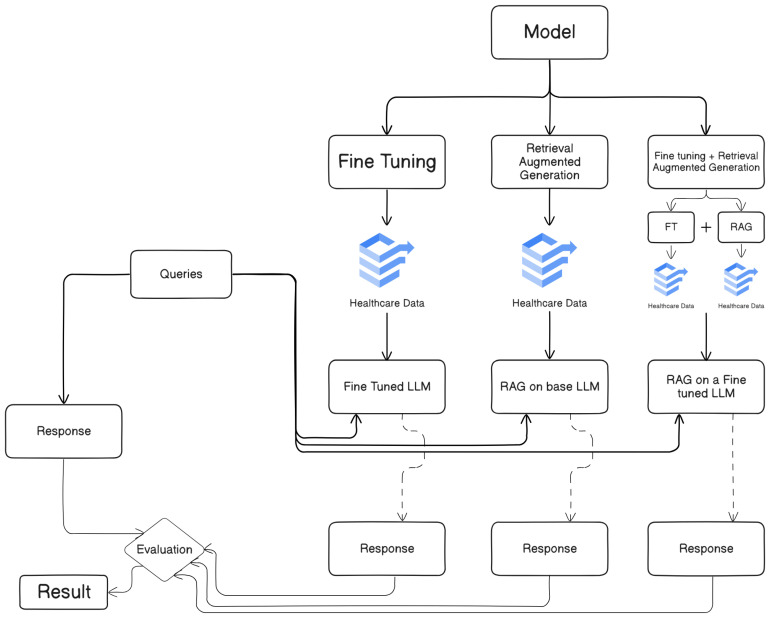
Proposed framework.

Standard healthcare-related searches were used to evaluate every model—fine-tuned, RAG applied to a standard LLM, and RAG applied to a fine-tuned model. Using evaluation criteria including BLEU, GLEU, ROUGE-1, ROUGE-2, ROUGE-L, METEOR, Precision, Recall, F1, BERTScore Precision, BERTScore Recall, BERTScore F1, SBERT Cosine Similarity, and Negation-Aware Semantic Similarity, the responses were evaluated in relation to reference answers. By means of a comparative analysis of these evaluation findings, the strengths and shortcomings of every method were exposed, thus guiding the most successful strategy for tasks connected to healthcare and choosing which model best fits the situation. The combined results provide insightful analysis concerning how well these approaches produce exact and contextually suitable responses in the healthcare field. The suggested framework of our model is shown in Figure 1.

## 4. System Design Architecture

The main components—the medical QA dataset, the selected LLMs, ChromaDB (which acts as the vector database for RAG and hybrid fine-tuning with RAG), and the system configuration—are described in this section.

### 4.1. Dataset

We used MedQuAD (Medical Question Answering Dataset) [21] as the baseline dataset for assessing our system for this work. MedQuAD is a large and well-organized dataset of 47,457 question–answer pairs taken from reputable medical sources, the National Institutes of Health (NIH), Genetics Home Reference, and ClinicalTrials.gov. The dataset contains a broad array of medical issues, including disease symptoms, differential diagnoses, treatment, medications, and genetic conditions, and hence is a highly trustworthy source to train and evaluate AI-based healthcare applications.

**Figure 2:**
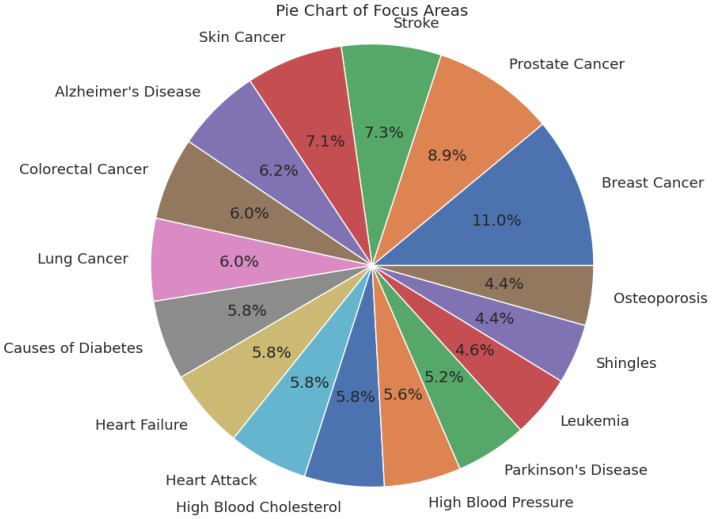
Pie chart of focus areas by health condition.

The MedQuAD dataset is spread across 5126 distinct medical focus areas, including a wide variety of conditions like Glaucoma, Diabetes, Shingles, Leukemia, and several others. The focus areas cut across different domains of medicine like neurology, cardiology, endocrinology, oncology, and infectious diseases, and cover clinically important topics in detail. In order to study the distribution of medical topic areas, we show a pie chart of the ratio of topics with more than 20 questions. Figure 2 displays the pie chart of different proportions of topics. MedQuAD’s formalized format and expert-checked answers make it especially well-suited for medical question-answering, enabling the creation and benchmarking of LLMs and retrieval-augmented generation systems. Using this dataset, we guarantee that our model is tested on clinically applicable queries, enabling an objective comparison of its accuracy, contextuality, and reasoning ability within the healthcare domain.

### 4.2. Large Language Models

We used the following LLMs in our research—Meta-Llama-3.1-8B, Phi-3.5-mini-instruct, Gemma-2-9B, Mistral-7B-Instruct, and Qwen2.5-7B—all of which were 4-bit quantized using the Unsloth [22] library to minimize memory usage when fine-tuning.

Meta-Llama-3.1-8B, created by Meta, is particularly renowned for its robust performance on instruction-following and reasoning tasks. Phi-3.5-mini-instruct, published by Microsoft, is a small but efficient model suited for cost-effective deployment, especially effective in instruction-tuned environments. Gemma-2-9B, designed by Google, is optimized for helpfulness and safety and hence can be applied to general-purpose conversational work. Mistral-7B-Instruct is optimized for speedy inference and good multitask performance and provides competitive speed and accuracy. Finally, Qwen2.5-7B, created by Alibaba, is designed for multilingual and domain-oriented tasks and specifically excels in dealing with long instructions and dialog-based interactions. All the models utilized in this work were accessed through the Unsloth library, which offered optimized versions to enable effective loading, quantization, and fine-tuning of LLMs. Unsloth is an open-source library for optimizing large language model fine-tuning. It improves training efficiency by offering two to five times faster performance and dramatically reduces memory usage—up to 80%. These optimizations make it a viable option for environments with sparse computational resources.

### 4.3. ChromaDB

We utilized ChromaDB as the vector database to enable the retrieval-augmented generation and fine-tuning + RAG (FT+RAG) methods. ChromaDB stores and indexes dense vector embeddings derived from the medical knowledge base efficiently, allowing for quick and accurate retrieval of relevant documents during inference. Its integration guarantees low-latency retrieval, which is essential for real-time response generation. ChromaDB was employed by [23], which showed how effective ChromaDB could be in semantic similarity estimation and information retrieval. Through the integration of ChromaDB and language models, the system was able to incorporate dynamic external information.

### 4.4. System Configuration

The models were fine-tuned on two different hardware configurations. GEMMA and PHI were trained on a system with a single Tesla V100-PCIE-32GB GPU, backed by eight physical CPU cores (eight logical cores) and 32GB of VRAM. Conversely, MISTRAL, QWEN, and LLAMA were fine-tuned with a more powerful system consisting of two NVIDIA GeForce RTX 4090 GPUs, 16 physical CPU cores (24 logical cores), and 24 GB of VRAM for each GPU. The wide difference in hardware resources had a direct impact on training efficiency and played a part in shorter fine-tuning times for models that had been trained on the RTX 4090 system.

## 5. Methodology

This section presents the comprehensive procedures in fine-tuning, retrieval-augmented generation, and their blend (FT+RAG) and how the evaluation framework for comparing the performance of these methods across language models is utilized. This comprises dataset preparation, model setup, training workflows, the incorporation of external knowledge through retrieval, and the metrics and protocols applied to gauge each method’s performance in the medical question-answering task.

### 5.1. Preprocessing of Dataset

Every record in the dataset was converted to the form suitable for training of the models, which are capable of learning from question–answer pair data. Subsequently, the dataset was restructured as well. For the case of doctor–patient interactions, the question was set as the patient’s inquiry and the response was assigned as the respective answer. The data was framed in the format of a prompt template containing instructions, inputs, and outputs based on the Alpaca dataset format. In order to receive correct text generation at inference, an End of Sequence (EOS) token was added to all examples.

**Figure 3:**
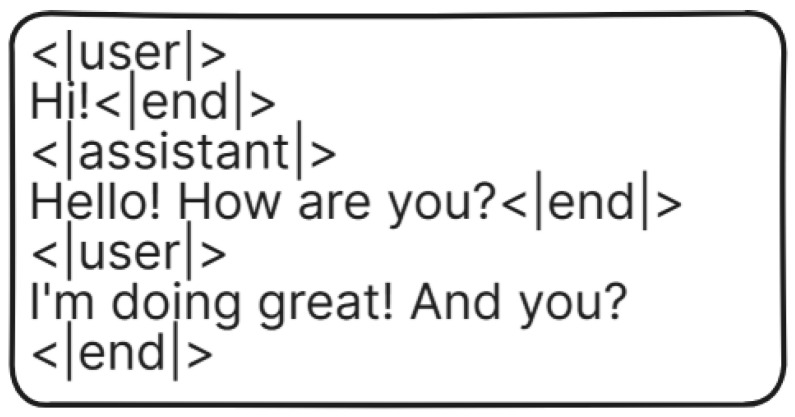
Phi-3 format used for conversation-style fine-tuning.

For fine-tuning PHI, we adopted the Phi-3 format for conversational data, utilizing Open Assistant conversations in the ShareGPT style. Phi-3 structures multi-turn conversations as shown in Figure 3.

**Figure 4:**
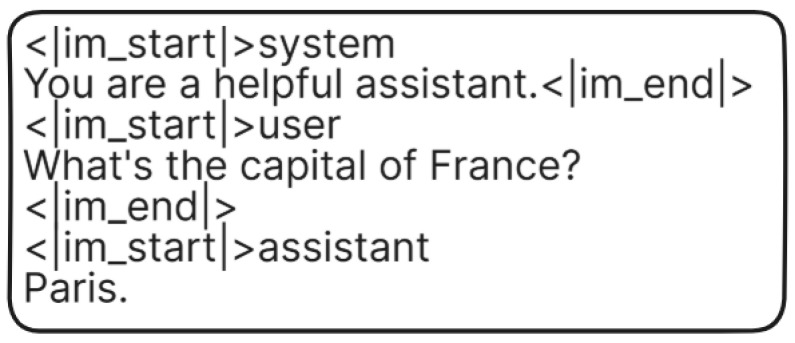
ChatML format used for conversation-style fine-tuning.

For MISTRAL, we employed the ChatML format for conversation-style fine-tuning, also leveraging Open Assistant conversations in the ShareGPT style. ChatML structures multi-turn interactions as shown in Figure 4.

**Figure 5:**
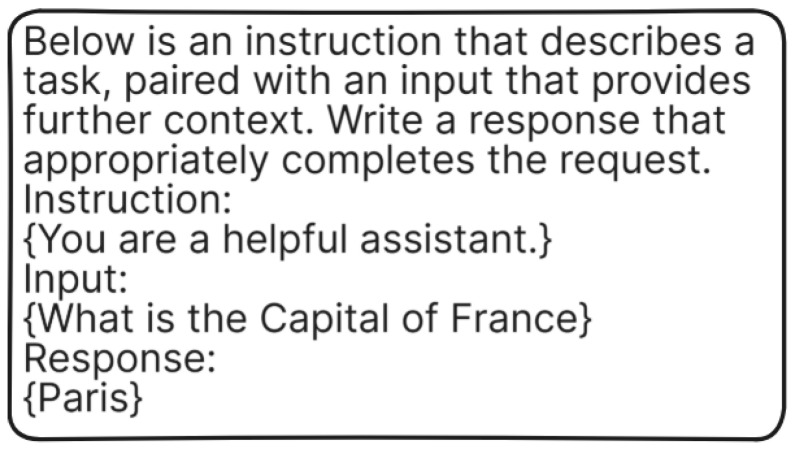
Alpaca Prompt format for conversation style fine-tuning.

In contrast, for LLAMA, GEMMA, and QWEN, we employed the Alpaca prompt format, as illustrated in Figure 5.

### 5.2. Fine-Tuning and RAG Implementation

Following pre-processing of the datasets, we carried out three different methods for improving the LLM’s performance: fine-tuning, RAG, and fine tuning + retrieval-augmented generation (FT+RAG).

#### 5.2.1. Fine-Tuning

The models were fine-tuned on an expanded, domain-specific dataset, MedQuAD (Medical Question Answering Dataset), which comprises 47,457 well-structured QA pairs obtained from reliable medical websites. It was used to refine the models. Every entry was restructured for training: MISTRAL followed ChatML; LLAMA, GEMMA, and QWEN adopted the Alpaca prompt style; and PHI used the Phi-3 conversational format. This second training stage consisted of tuning the model weights and biases to refine its capacity for generating precise, contextually correct answers to clinical questions. To speed up computations, the model was quantized at 4-bit precision using the BitsAndBytes Python library. Training was over a single epoch, with every entry in each dataset converted to a predefined template optimized for training an LLM. The template used here had two major elements: the medical question was included in the first part, and the second included the respective answer. This formatted process allowed the model to learn properly from the dataset so that it could produce high-quality medical replies.

Large language model fine-tuning in this research was maximized using various libraries to achieve efficiency and lower computing expenses. Unsloth was used to speed up the fine-tuning process while keeping memory usage low. Transformers, a library offered by Hugging Face, allowed for using pre-trained models and tokenizers. Datasets, a library also offered by Hugging Face, was utilized for seamless dataset loading and preprocessing. To make better use of memory, BitsAndBytes was used to support 4-bit quantization, which lowered computational overhead during training. Xformers was also used to implement efficient attention, enhancing training speed.

For parameter-efficient fine-tuning, Parameter-Efficient Fine-Tuning (PEFT) [24] was used, with techniques like Low-Rank Adaptation (LoRA) [7]. TRL (Transformer Reinforcement Learning), another library of Hugging Face, was used for supervised fine-tuning (SFT) and reinforcement learning with transformers. SentencePiece was used for tokenization and text processing. Hugging Face Hub was used at last for interacting with Hugging Face’s model repository, with the ability to store and share models easily. QLoRA (Quantized Low-Rank Adaptation) [8] is a memory-efficient way of fine-tuning LLMs with low-rank adaptation on quantized weights, which saves a lot of memory while maintaining performance. It allows running and training big models on consumer-grade GPUs by mixing 4-bit quantization with parameter-efficient tuning.

Fine-tuning settings: The model was fine-tuned with LoRA, a parameter-efficient fine-tuning method that modifies only a proportionate (1–10%) small subset of the model parameters through the addition of low-rank matrices to certain layers. The LoRA setup had the following:

- Rank (r): 16, which controlled the number of parameters in the low-rank matrices.
- Target Modules: Used on query, key, value, and output projection layers.
- Gradient Checkpointing: Used to limit the use of memory when training.

Fine-tuning was achieved with Hugging Face’s SFTTrainer from the Transformer Reinforcement Learning (TRL) library. The arguments for training were specified as follows:

- Batch size: 2 (per device).
- Gradient accumulation steps: 4.
- Learning rate: 2 × $10^{-4}$.
- Optimizer: AdamW with 8-bit precision.
- Mixed precision training: FP16.

The model was adapted through LoRA through FastLanguageModel.get_peft_model() with a rank of 16 and LoRA alpha at 16, used on key projection layers (q_proj, k_proj, v_proj, o_proj, gate_proj, up_proj, down_proj). Dropout and bias were disabled for optimal performance in the configuration. Gradient checkpointing (“unsloth”) was enabled, saving VRAM by 30%.

Stabilized LoRA was supported, and LoftQ quantization was excluded. A random seed was set to provide reproducibility. Moreover, the “unsloth” backend was utilized to accelerate efficiency, specifically for long-context operations. The Alpaca-style prompt template organized inputs into instruction, context, and expected output fields. A format function operated on examples by joining these fields together with the template, adding EOS tokens to denote sequence boundaries, and returning batches of formatted text. The data was obtained from MedQuAD. Prompt formatting was performed during preprocessing, with batch processing turned on for optimization. Special treatment involved imposing EOS tokens to avoid generating text infinitely and preserving structured output to guarantee dialogue coherence.

The SFTTrainer was set up for supervised fine-tuning on the preprocessed MedQuAD dataset. Training hyperparameters were a batch size of two per device, four gradient accumulation steps, five warmup steps, and a single epoch with a learning rate of 2 × $10^{-4}$. Optimization was achieved using 8-bit AdamW with linear learning rate scheduling and a weight decay of 0.01. Mixed-precision training was activated through fp16 or bf16, depending on hardware support. Logging was carried out at every step, and results were monitored using Weights & Biases (wandb). The selected hyperparameters were adopted from prior studies [25,26,27], as they employed the same Unsloth library. These configurations facilitated rapid and resource-efficient model training.

The model and the tokenizer were saved on the local system in a merged 16-bit format, finding a balance between precision and storage. The trained model was then pushed to the Hugging Face Hub in the same 16-bit merged format to maximize deployment. During fine-tuning, the model was trained with 4-bit precision via QLoRA, which effectively cut the GPU memory footprint by about half while maintaining performance. Nonetheless, to enable inference and deployment, the model was also back-translated to 16-bit for improving numerical stability and output quality over 4-bit. Further, 16-bit precision is widely available across GPUs and TPUs without special-purpose kernels, providing a better balance between accuracy and memory efficiency during inference.

#### 5.2.2. Retrieval-Augmented Generation (RAG)

In this method, the models are provided with an external retrieval system. At the time of response generation, the model fetches information from a curated knowledge base to enhance its pre-trained and fine-tuned knowledge. The information retrieved is then combined with the internal knowledge of the model, enhancing its capacity to generate rich and current responses.

The pipeline starts with transforming the training dataset into a structured pandas DataFrame and saving it as a CSV file for normalized processing. Document chunks are produced through a recursive text splitter with a 500-token window that optimizes retrieval granularity. To enable efficient inference, we preload the LLMs in 4-bit in conjunction with all-MiniLM-L6-v2 sentence embeddings, which encode document chunks for semantic retrieval. The text-generation pipeline uses top-k sampling (k = 30) with temperature 0.5 to trade response diversity and coherence, limiting outputs to 256 tokens. For RAG, optimal performance was achieved with a top-k below 30 [28], as higher values reduced efficiency. Temperature settings were tuned to balance randomness and precision—lower values (e.g., 0.5) prioritized high-probability tokens, while higher values introduced greater variability [29,30]. An efficient similarity search over the embedded chunks is supported by a Chroma vector database, and a memory-free ConversationalRetrievalChain queries the database (fetching the most relevant chunk per question) and produces responses through the LLMs. This stateless RAG architecture guarantees isolated, context-independent answers. The blend of 4-bit inference and optimized retrieval provides computational efficiency without any loss of output quality, thus making the pipeline appropriate for large-scale deployment in medical question-answering applications.

#### 5.2.3. Retrieval-Augmented Generation on Fine-Tuned Models (FT+RAG)

In the third approach, RAG was used on the fine-tuned models, taking the best from both methods to maximize performance. The hybrid FT+RAG approach reused the same fine-tuned models from the FT setup, with RAG applied using identical settings. In fine-tuning, the models were again trained on a larger, specialized dataset of medical situations. This fine-tuning tuned the model’s weights and biases to enhance its contextual, accurate, and specialized responses to medical questions. In the RAG method, the fine-tuned models were combined with a retriever system that allowed them to retrieve information from an external, pre-curated knowledge database during response generation. The retrieved data was added to the model’s fine-tuned and pre-trained knowledge, allowing it to generate more extensive, contextually appropriate, and current responses.

### 5.3. Evaluation Using a Query Set

To comparatively evaluate the effectiveness of fine-tuning and retrieval-augmented generation and FT+RAG objectively, an evaluation protocol was developed based on 75 standardized medical questions. These questions were meticulously selected to mimic real-world medical questions about disease symptoms, differential diagnoses, and possible treatment. This evaluation protocol acted as a thorough benchmark for measuring the strengths and weaknesses of fine-tuning and RAG in delivering reliable and contextually relevant information to assist medical professionals and patients.

#### Evaluation Metrics

To assess the models’ performance, a wide range of metrics was used, reflecting both surface and semantic similarities between generated and reference text. BLEU (Bilingual Evaluation Understudy) was used as a precision-based metric to assess n-gram overlap between the generated and reference outputs with a penalty for responses too short. Although it was extensively used in machine translation activities, it did not completely capture semantic adequacy. To overcome this drawback, GLEU (Google-BLEU) was also applied, which included both recall and precision, providing a balanced assessment. Token-level classification metrics were used as well. Precision measured the percentage of relevant tokens that were brought back by the model, whereas Recall estimated the percentage of actual relevant tokens that were tagged correctly. Their harmonic mean, the F1 measure, captured the trade-off between them.

Our research also utilized the ROUGE family of measures. ROUGE-1 and ROUGE-2 calculated unigram and bigram overlap, respectively, indicating lexical similarity. ROUGE-L computed the longest common subsequence between texts, indicating sentence-level structural similarity. METEOR was also used to match generated and reference texts on exact matches, stemming, synonyms, and paraphrasing. METEOR traded precision and recall, thus offering more semantically aware assessment.

For semantic similarity evaluation at a more profound level, context embedding-based measures were utilized. BERTScore, which was based on BERT embeddings to compare token representations, was calculated in terms of precision (BERTscore Precision), recall (BERTscore Recall), and F1 (BERTscore F1) to capture different aspects of similarity. BERTScore Precision assesses how semantically similar the predicted tokens are to the reference tokens using contextual embeddings. BERTScore Recall assesses how well the predicted tokens capture the meaning of the reference tokens. The harmonic mean of BERTScore Precision and Recall is BERTScore F1. This gave a balanced measure of how well the two words fit together semantically. SBERT Cosine Similarity was applied to evaluate the cosine similarity of sentence embeddings generated by SBERT, providing a strong evaluation of semantic matching at the sentence level. We computed the Negation-Aware Semantic Similarity (NASS) score as the cosine similarity between sentence embeddings generated using a model fine-tuned to capture semantic shifts caused by negation, specifically the all-mpnet-base-v2-negation model (fine-tuned model within the context of [31]). The evaluation metrics employed in this study were chosen due to the fact that they are among the most widely used criteria for evaluating LLMs’ [32] performance in the medical field.

## 6. Results and Discussion

This section showcases the results of our work, which encompass training performance of several LLMs, comparative evaluation of their perplexity scores, and lexical and semantic evaluations based on various metrics. It also examines the advantages and limitations of every model and adaptation approach (fine-tuning, RAG, and FT+RAG) leading to concluding with key findings summarizing the most optimal strategies for medical question-answering tasks. Lastly, we extensively discuss a comprehensive comparison between our model and other models out there, pointing out important differences and similarities.

### 6.1. Model Training Outcomes

This section explores the model training results, such as training time, training loss, GPU energy consumption, and GPU performance.

#### 6.1.1. Training Duration

The total fine-tuning durations recorded for each model were as follows: GEMMA took 3 h, 4 min; PHI required 3 h, 13 min; MISTRAL completed in 1 h, 20 min; QWEN in 1 h, 15 min; and LLAMA in 1 h, 19 min. GEMMA and PHI models were trained on an eight physical and eight logical CPU cores, a single Tesla V100-PCIE-32GB GPU, and 32 GB of VRAM setup. In comparison, MISTRAL, QWEN, and LLAMA were trained on a more robust configuration with 16 physical and 24 logical CPU cores, dual NVIDIA GeForce RTX 4090 GPUs, and 24 GB of VRAM, which accounted for their relatively shorter training times.

#### 6.1.2. Train/Loss

**Figure 6:**
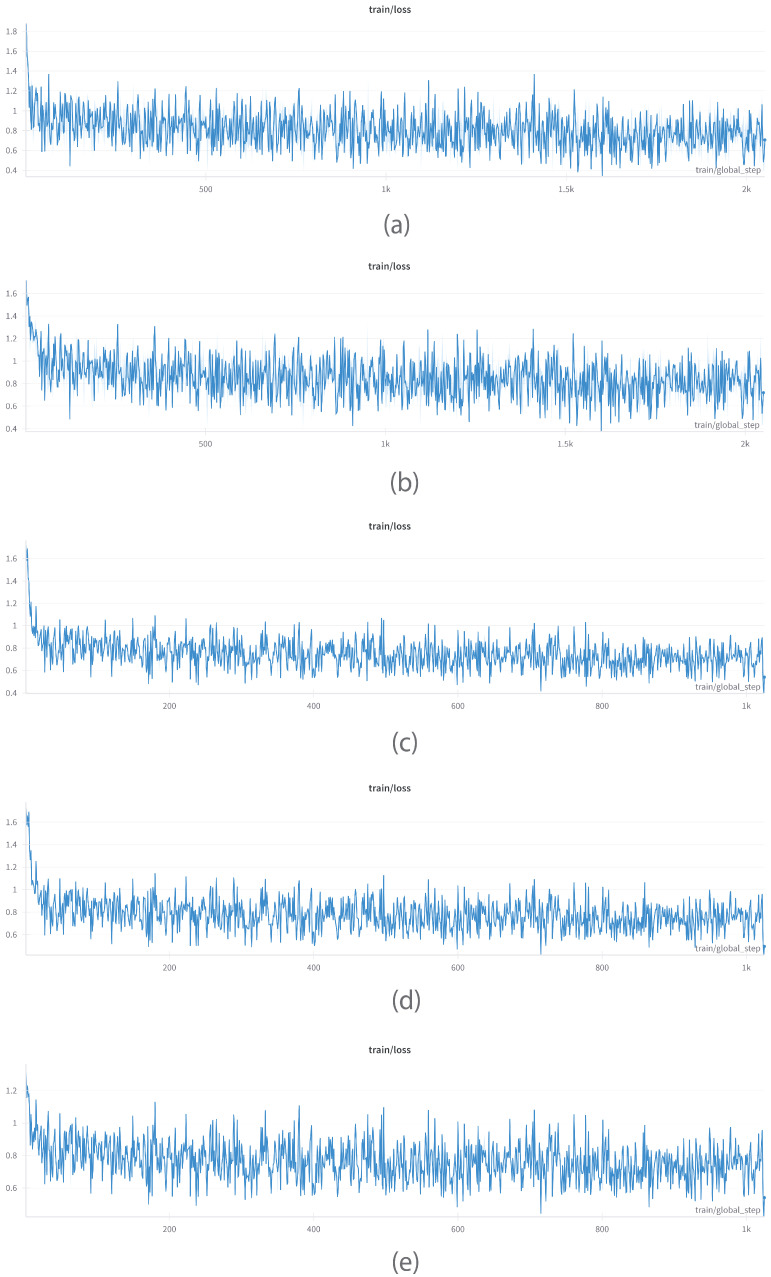
Train/loss over time graph for (**a**) GEMMA, (**b**) PHI, (**c**) MISTRAL, (**d**) LLAMA, and (**e**) QWEN.

Figure 6 shows training loss curves for five language models—GEMMA, PHI, MISTRAL, LLAMA, and QWEN—plotted as a function of training steps. All models have an early sharp loss reduction, indicating fast initial learning. But the level of stability and convergence is different across them. GEMMA (Figure 6a) and PHI (Figure 6b), both trained on a single Tesla V100 GPU, show significant fluctuations during training. Their high-frequency fluctuations indicate instability in the learning process despite a general declining trend. This can be attributed to hardware constraints affecting batch size, memory, and training throughput, resulting in noisy optimization.

Conversely, MISTRAL (Figure 6c), LLAMA (Figure 6d), and QWEN (Figure 6e) were optimized with two RTX 4090 GPUs, enabling more parallelism and bigger batch sizes during training. These models exhibit more regular and stable training dynamics. MISTRAL demonstrates a smoother loss curve with smaller variance and consistent dropping, reflecting successful optimization. QWEN has the most stable loss curve among all models, with very little oscillation after the initial decline, indicating highly efficient convergence. LLAMA shows a balanced trend, with a steady downward slope and moderate noise. In general, whereas all models overlap successfully, running more powerful equipment in MISTRAL, LLAMA, and QWEN generates more stable and efficient training that clearly separates these from the more erratic patterns characteristic of GEMMA and PHI.

#### 6.1.3. GPU Power Consumption

**Figure 7:**
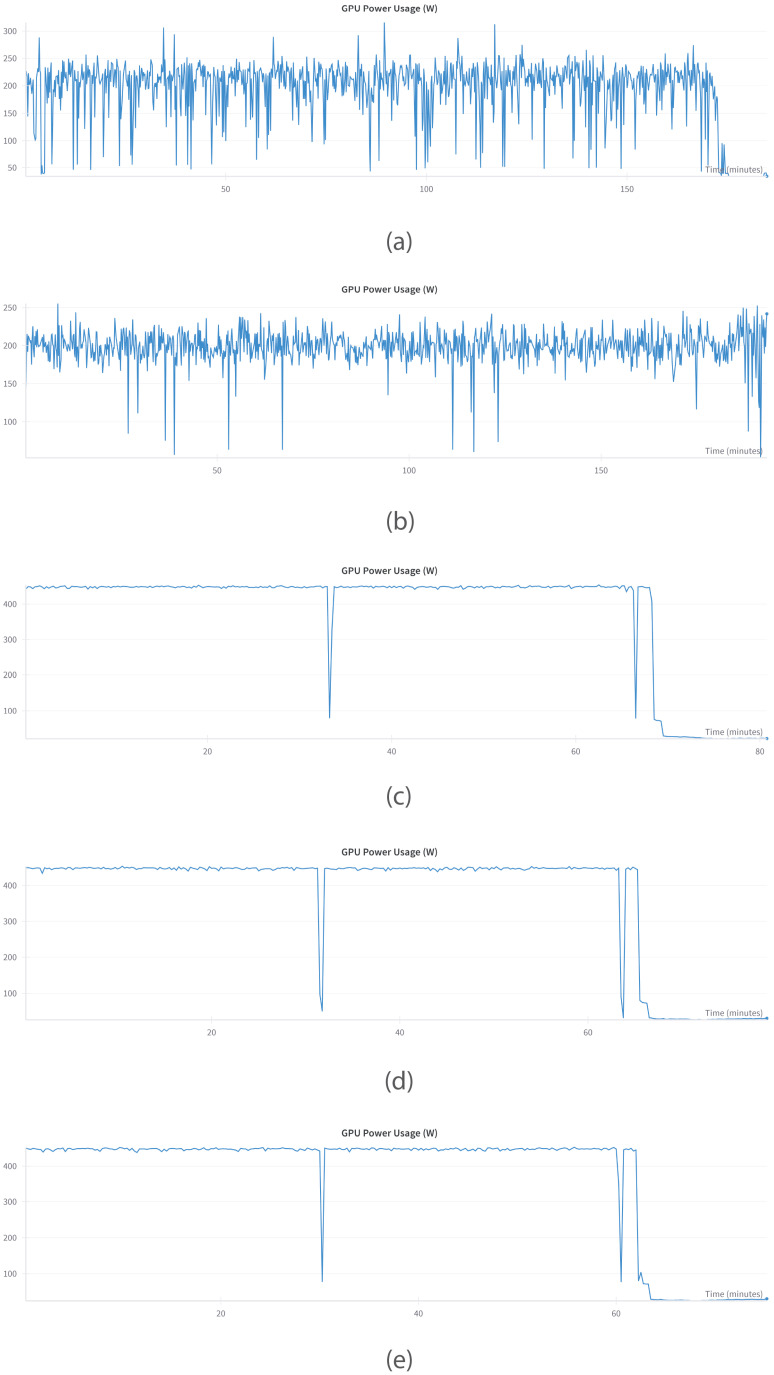
GPU power consumption over time showing variations in efficiency and workload behavior for (**a**) GEMMA, (**b**) PHI, (**c**) MISTRAL, (**d**) LLAMA, and (**e**) QWEN.

Figure 7 demonstrates GPU power usage patterns for five language models—GEMMA, PHI, MISTRAL, LLAMA, and QWEN—emphasizing variations in computational behavior throughout model runtimes. GEMMA (Figure 7a) and PHI (Figure 7b) have very dynamic power consumption, with high fluctuations between around 150–250 W. These fluctuations point to less regular GPU usage, possibly caused by sporadic workloads or architectural features that cause variable processing needs. Although PHI is slightly more stable than GEMMA, both indicate phases of operation with idle or underused GPU states. These models were trained on an NVIDIA Tesla V100 GPU, which might also affect the power consumption patterns because of differences in architecture, thermal limits, and memory bandwidth from newer consumer-grade GPUs.

In contrast, MISTRAL (Figure 7c), LLAMA (Figure 7d), and QWEN (Figure 7e) exhibit a dramatically different power profile. These models show a long, high-power usage plateau at around 400 W throughout their active periods with periodic brief drops during initialization and termination. This steadiness indicates effective GPU saturation and optimized workloads with negligible idle periods. The training of MISTRAL, LLAMA, and QWEN was performed on two NVIDIA RTX 4090 GPUs. The larger memory capacity and higher computational performance of the RTX 4090s contribute to the measured power stability and high utilization, supporting efficient parallelization and maximal use of hardware resources. Generally, the comparison highlights a compromise between variable vs. stable GPU utilization, the latter pointing towards better efficiency and optimization in model training and deployment environments.

#### 6.1.4. GPU Performance

The GPU usage patterns of five LLMs, namely, GEMMA, PHI, MISTRAL, LLAMA, and QWEN, exhibit unique computational patterns when in use.

**Figure 8:**
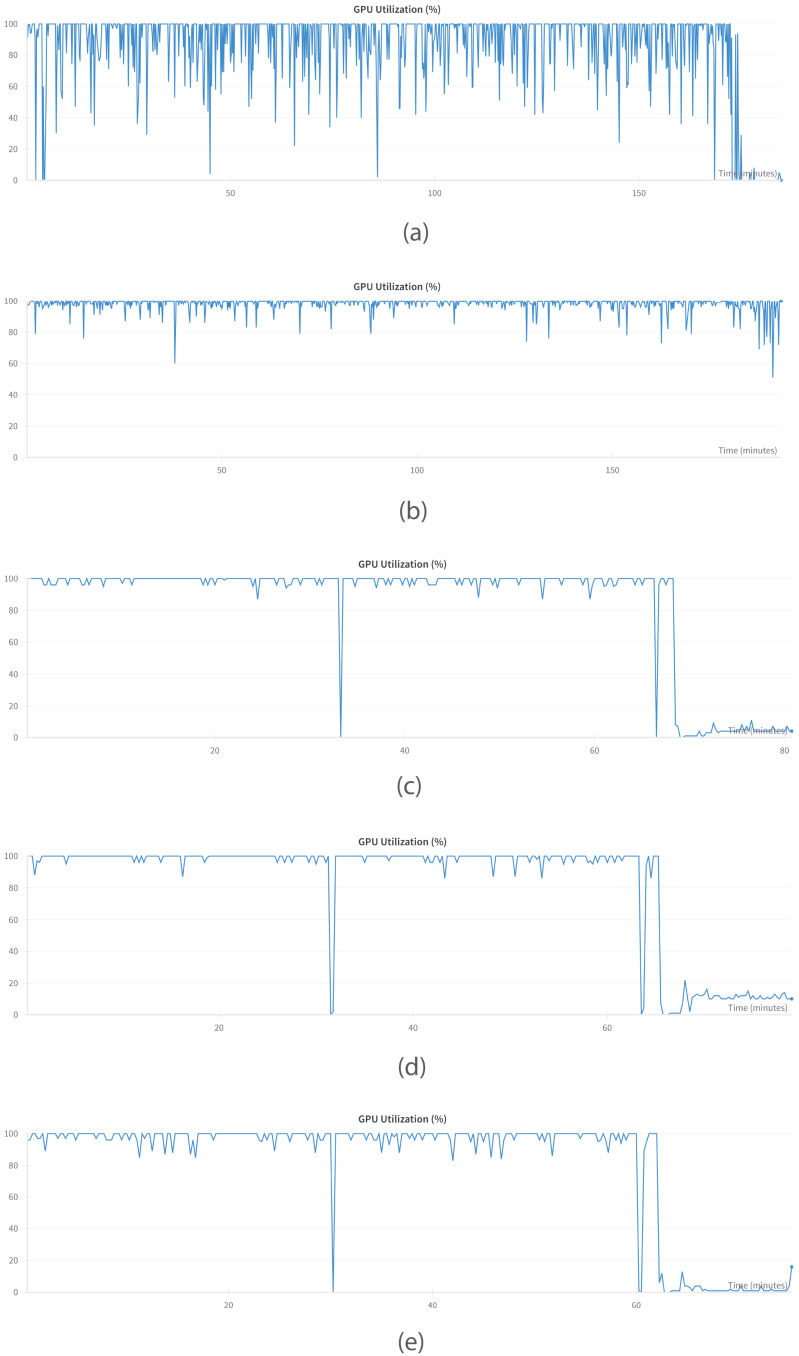
GPU utilization over time showing variations in efficiency and workload behavior for (**a**) GEMMA, (**b**) PHI, (**c**) MISTRAL, (**d**) LLAMA, and (**e**) QWEN.

Figure 8 illustrates the GPU usage patterns during the training process, demonstrating the computational workload allocation for every LLM. GEMMA (Figure 8a) and PHI (Figure 8b), both trained on one NVIDIA Tesla V100 GPU, have different profiles. GEMMA displays extremely irregular usage, with numerous and abrupt dips to 0% and spikes to 100%, which indicates processing or data handling inefficiencies creating uneven workloads. In contrast, PHI shows the most stable and consistent GPU usage, holding itself at near 100% for the entire duration of its execution with very few dips, demonstrating optimal resource management and prolonged processing.

MISTRAL (Figure 8c), LLAMA (Figure 8d), and QWEN (Figure 8e) were executed on machines with dual NVIDIA RTX 4090 GPUs, providing much higher throughput and memory bandwidth. All three models exhibit comparable patterns, with long stretches of high GPU usage with occasional rapid spikes downwards—presumably from data loading, synchronization, or checkpointing—and a significant drop in activity towards the end, indicative of a shift from heavy computation to post-processing or less demanding activity. Whereas all three tend to be productive in their operational core phases, their end phase behavior indicates planned execution with distinguishable workload phases. In total, PHI emerges as the stable performer in efficiency on the Tesla V100, whereas MISTRAL, LLAMA, and QWEN exploit the high-end dual RTX 4090 configuration well, with GEMMA pointing out likely optimization areas.

### 6.2. Perplexity as a Benchmark for LLMs

Among the evaluated models, PHI produced the least perplexity (PPL) value of 33.64 when fine-tuned, displaying exceptional robustness under 4-bit quantization for medical applications. QWEN and LLAMA presented relatively modest PPL values of ~40–50, in which LLAMA showed appreciable improvement post-fine-tuning. A few models suffered quite badly with 4-bit quantization. MISTRAL, for example, produced a very high PPL of 186.43 prior to fine-tuning, which did not reduce at all by way of post-fine-tuning down to merely 86.79, indicating rather extreme deterioration in performance. This indicates that MISTRAL has difficulty with medical terminology under aggressive quantization. Likewise, MISTRAL’s PPL rose following fine-tuning by 8.11, which may be a sign of overfitting or poor hyperparameter tuning. The effect of fine-tuning was different across models.

Fine-tuning effectiveness is highly dependent on the model’s pretraining quality and its ability to adapt to domain-specific data. MISTRAL’s performance points to the dangers of forceful quantization, specifically for domain-specific tasks, where careful adjustment is needed. In medical QA applications, 4-bit PHI provides, according to perplexity scores, the optimal balance between performance and efficiency. Table 2 presents the perplexity scores pre- and post-fine-tuning with the MedQuAD dataset, illustrating the effect of domain adaptation on language modeling performance.

### 6.3. Lexical and Semantic Evaluation (Quantitative Results)

The test compared five LLMs—GEMMA, PHI, QWEN, LLAMA, and MISTRAL—over three fine-tuning techniques: fine-tuning (FT), retrieval-augmented generation (RAG), and an aggregated fine-tuning + RAG (FT+RAG) method. Performance was tested over various assessment metrics, from the classical NLP metrics (BLEU, GLEU, ROUGE, METEOR) to precision–recall statistics and newer semantic similarity scores (BERTScore, SBERT cosine similarity, Negation-Aware Semantic Similarity). Table 3 presents an array of evaluation metrics—such as ROUGE, BLEU, METEOR, BERTScore, and more—which have been employed to evaluate model responses under FT, RAG, and FT+RAG configurations.

GEMMA proved competitive on several assessment criteria. Using the RAG approach, the model outperformed its fine-tuned (FT) counterpart, which scored 0.048 and 0.129, respectively, with a BLEU score of 0.123 and a GLEU score of 0.190. With a BLEU score of 0.104 and a GLEU score of 0.181, the hybrid FT+RAG approach produced intermediate results. With ROUGE-1 scores of 0.354 (RAG) against 0.313 (FT), RAG regularly exceeded FT in terms of ROUGE measures. Indicating better alignment with reference texts, the METEOR score likewise increased from 0.196 (FT) to 0.235 (RAG). These findings imply that RAG improves GEMMA’s text generating capacity, although the hybrid approach did not always outperform standalone RAG.

PHI showed especially better results using RAG and hybrid techniques. Among all configurations, the FT+RAG method attained the highest BLEU (0.136) and GLEU (0.217) scores, exceeding both FT (0.036, 0.122) and standalone RAG (0.111, 0.190). With FT+RAG scoring ROUGE-1 of 0.391, much higher than FT (0.267) and RAG (0.366), ROUGE measures followed a similar trend. For FT+RAG, the METEOR score likewise peaked at 0.258, suggesting better semantic alignment. These results show PHI’s capacity to efficiently combine fine-tuning with retrieval-augmented generation, thus producing the best overall performance.

**Figure 9:**
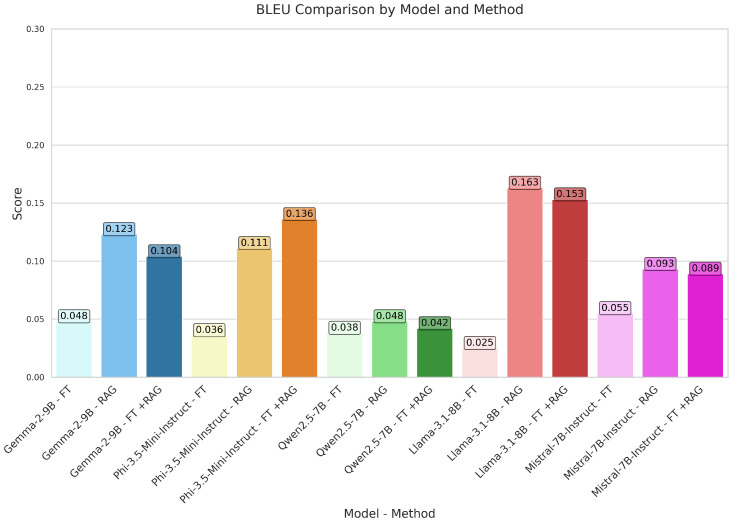
Model-wise BLEU score analysis: fine-tuning, RAG, and FT+RAG approaches.

**Figure 10:**
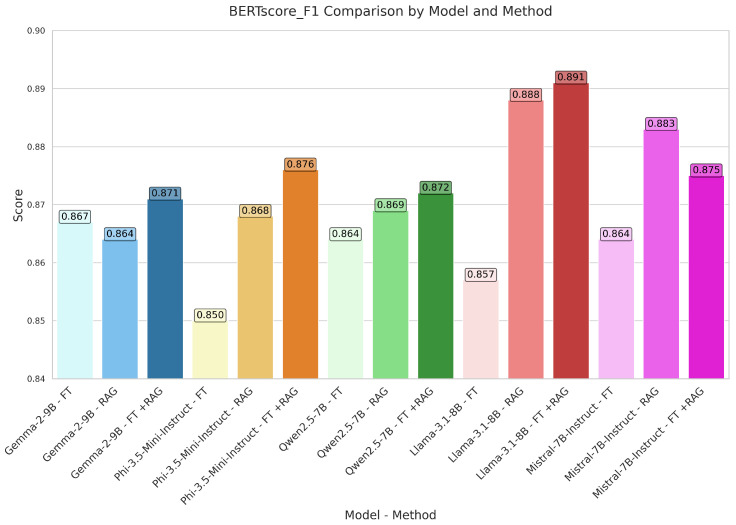
Model-wise BERTScore F1 evaluation: fine-tuning, RAG, and FT+RAG methods.

Under several evaluation configurations, QWEN produced conflicting results. While RAG and FT+RAG produced only slight improvements (0.048 and 0.042, respectively), the FT approach scored a BLEU of 0.038. GLEU scores showed a similar trend; RAG (0.128) somewhat exceeded FT (0.109). Though the increases were little, ROUGE-1 scores for RAG (0.306) and FT+RAG (0.302) were higher than FT (0.276). Over all configurations, the METEOR score stayed rather low; it peaked at 0.161 for RAG, implying little change in semantic coherence. Generally, RAG or hybrid techniques did not help QWEN perform as much as other models. Figure 9 illustrates the BLEU scores achieved by our models across different methods. Figure 10 highlights the BERTScore F1 scores, integrating both precision and recall.

Strong performance was given by LLAMA, especially with RAG and FT+RAG methods. Among all configurations, the RAG approach obtained the best BLEU score (0.163); FT+RAG followed rather closely (0.153). With RAG (0.237) and FT+RAG (0.236) outperforming FT (0.089), GLEU scores were likewise rather competitive. ROUGE measurements underlined this benefit even more; RAG and FT+RAG obtained ROUGE-1 scores of 0.418 and 0.423, respectively, compared to 0.262 for FT. Additionally indicating excellent semantic alignment, the METEOR score peaked at 0.279 for RAG. When combined with retrieval-based techniques, these results place LLAMA among the best models.

Different performance was shown by MISTRAL based on the evaluation configuration. While RAG (0.093) and FT+RAG (0.089) showed only modest gains, the FT approach obtained a BLEU score of 0.055. GLEU scores showed a similar pattern; RAG (0.186) outperformed FT (0.136). Though FT+RAG (0.346) did not match this performance, RAG (0.386) received highest ROUGE-1 ratings. With RAG, the METEOR score peaked at 0.220, implying better semantic coherence than with FT (0.206). Although RAG improved MISTRAL’s performance, the hybrid FT+RAG approach did not routinely outperform standalone RAG, suggesting possible trade-offs in method integration.

Semantic evaluation based on BERTScore and SBERT cosine similarity exposes subtle information on the performance of the models. Across all models, BERTScore precision was consistently high; LLAMA obtained the highest score (0.925 for FT+RAG), indicating strong token level alignment with reference texts. Though values stayed strong, especially for LLAMA (0.862 for RAG) and PHI (0.852 for FT+RAG), BERTScore recall showed more variability, suggesting good capture of important semantic content. With LLAMA (0.891 for FT+RAG) and PHI (0.876 for FT+RAG) leading, highlighting their balanced precision–recall performance, the harmonic mean confirmed these trends further. Complementing these findings with SBERT cosine similarity, which gauges contextual embedding alignment, LLAMA (0.822 for RAG) and PHI (0.815 for FT+RAG) once more showed exceptional semantic coherence at the sentence level.

**Figure 11:**
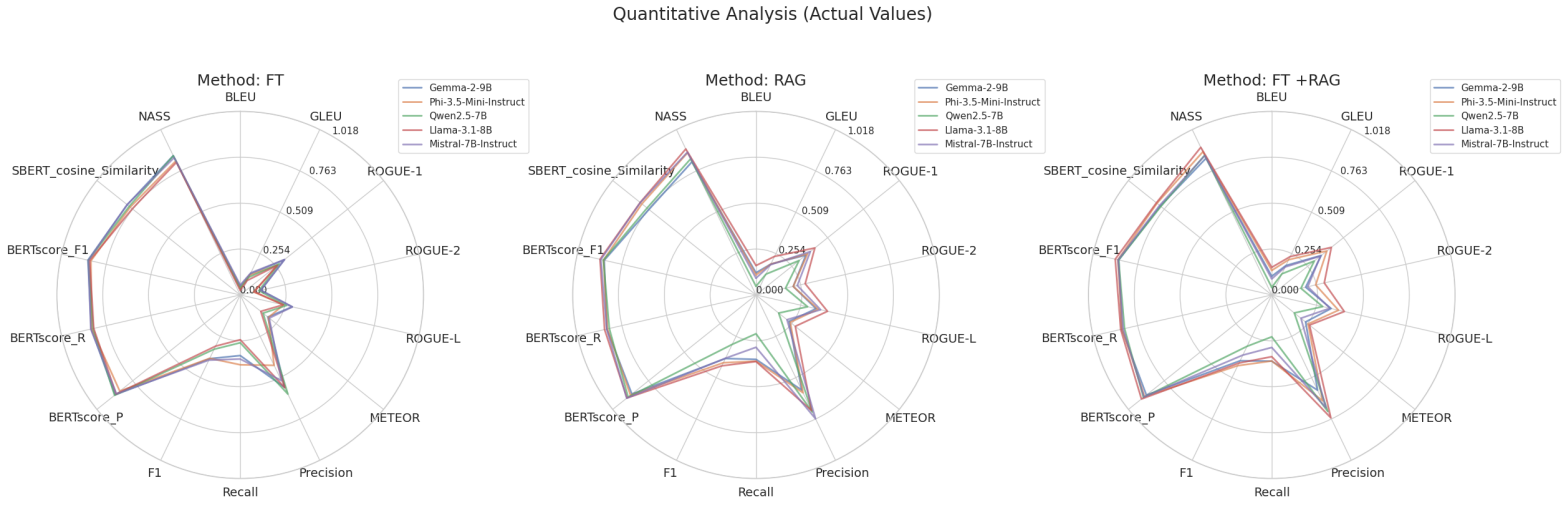
Radar plots comparing model performance across FT, RAG, and FT+RAG methods.

QWEN and MISTRAL showed less improvement even if most models gained from RAG or hybrid approaches in BERTScore measures. Though its SBERT similarity (0.787) lagged behind, implying less contextual alignment, QWEN’s BERTScore F1 peaked at 0.872 (FT+RAG). Despite a high BERTScore Precision (0.918 for RAG), MISTRAL showed a decrease in SBERT similarity for FT+RAG (0.796), suggesting possible trade-offs between token-level precision and sentence-level coherence. These results highlight how, although retrieval augmentation usually improves semantic fidelity, the degree of improvement varies depending on the model; LLAMA and PHI show the most consistent gains across both token- and sentence-level measurements. Figure 11 shows radar plots for every model, which allow for a visual understanding of their relative strengths under different adaptation approaches.

The Negation-Aware Semantic Similarity values across models exhibited significant patterns. Fine-tuning achieved the best NASS score of 0.857 for GEMMA, beating both RAG (0.819) and FT+RAG (0.841). On the other hand, PHI did the best with the hybrid FT+RAG method (0.881), followed closely by RAG (0.875) and FT (0.824). There were only small differences in QWEN, with FT+RAG coming in first at 0.859, FT coming in second at 0.856, and RAG coming in third at 0.837. LLAMA showed a clear benefit from augmentation, as FT+RAG (0.908) and RAG (0.898) did better than FT (0.816). Finally, MISTRAL did very well on RAG (0.878), then FT+RAG (0.857), and finally FT (0.845). This shows that RAG is better at capturing semantic similarity with negation.

### 6.4. Statistical Analysis

Bootstrapping with resampling to find 95% confidence intervals for score uncertainty was used. Bootstrap samples were generated with replacement to estimate the mean score distribution for each metric and method. Table 4 shows the 95% confidence intervals for evaluation metrics across all models and methods (FT, RAG, FT+RAG), restricted to only those cases where at least one pairwise comparison between methods was statistically significant (*p* < 0.05), as reported in Table 5. Metrics without significant differences are marked with “—” to avoid misleading interpretation. These intervals, based on a bootstrapped sample meant over the test questions, provided a statistical range within which the true metric values were likely to fall. In most cases, RAG and FT+RAG had higher upper and lower bounds than FT, especially in important metrics like BLEU, ROUGE, and BERTScore. In some cases, such as LLAMA and PHI, the intervals for RAG and FT+RAG are entirely above those of FT, reinforcing their statistical superiority.

We applied the Wilcoxon signed-rank test on per-question metric scores to see if the differences in performance between the methods (FT, RAG, FT+RAG) were statistically significant. This non-parametric test is ideal for paired data that do not follow a normal distribution. For each model and metric, we conducted three pairwise comparisons: FT vs. RAG, FT vs. FT+RAG, and RAG vs. FT+RAG, using a significance threshold of *p* < 0.05. Table 5 shows the statistical significance (*p*-values) for comparing the FT, RAG, and FT+RAG methods.

In GEMMA, RAG did much better than fine-tuning on 4 out of 14 metrics (BLEU, GLEU, ROUGE-2, and SBERT Cosine Similarity), which means that there were some improvements but not many. FT+RAG was only significant in one metric (GLEU), and RAG vs. FT+RAG was only significant in one metric (SBERT Cosine Similarity). In general, RAG had only small advantages over FT, and the hybrid method did not add much value.

For PHI, RAG did much better than FT in 11 out of 14 metrics (BLEU, GLEU, ROUGE-1, ROUGE-2, ROUGE-L, METEOR, Precision, BERTScore Precision, BERTScore Recall, BERTScore F1, NASS). For the PHI model, FT+RAG did better than FT in 12 metrics (BLEU, GLEU, ROUGE-1, ROUGE-2, ROUGE-L, METEOR, Precision, F1, BERTScore Precision, BERTScore F1, SBERT Cosine Similarity, NASS). FT+RAG was better than RAG in four metrics (GLEU, BERTScore P, BERTScore R, BERTScore F1), which suggests that the hybrid approach made small but steady improvements in meaning.

In QWEN, RAG did much better than fine-tuning on 4 out of 14 metrics (ROUGE-2, Precision, Recall, BERTScore Precision). In contrast, FT+RAG did better than FT on four metrics (ROUGE-2, Precision, BERTScore Precision, BERTScore F1). But none of the metrics showed that RAG and FT+RAG were statistically different from each other. These results indicate that both RAG and FT+RAG provide only slight enhancements over FT, with their benefits being limited and largely equivalent, showing no distinct superiority between the two.

When using LLAMA, both RAG and FT+RAG did much better than FT on all 14 evaluation metrics: BLEU, GLEU, ROUGE-1, ROUGE-2, ROUGE-L, METEOR, Precision, Recall, F1, BERTScore Precision, BERTScore Recall, BERTScore F1, SBERT Cosine Similarity, and NASS. This shows that the gains were strong and consistent. Nonetheless, no metrics demonstrated significance between RAG and FT+RAG, indicating that RAG alone is adequate for optimal performance.

RAG did much better than FT in 9 out of 14 metrics (ROUGE-1, ROUGE-2, ROUGE-L, Precision, Recall, BERTScore Precision, BERTScore F1, SBERT Cosine Similarity, NASS) for MISTRAL. FT+RAG, on the other hand, did better than FT in five metrics (ROUGE-2, Precision, Recall, BERTScore Precision, BERTScore F1). There were only three metrics that were significant when comparing RAG to FT+RAG: Precision, BERTScore Precision, and BERTScore F1. This means that FT+RAG only offers small improvements, and RAG alone is a strong baseline.

### 6.5. Key Findings

Our study answered four main questions: (1) Which model is best overall? (2) Which approach is best overall? (3) Which model is best for each approach? (4) Which approach is best for each model?

The comprehensive evaluation reveals that LLAMA comes out as the best performing model on most criteria. Along with great performance in conventional metrics like BLEU (0.163 for RAG) and ROUGE-1 (0.423 for FT+RAG), it achieves the best marks in critical semantic measures including BERTScore F1 (0.891 for FT+RAG) and SBERT cosine similarity (0.822 for RAG). PHI also shows good results, especially with hybrid approaches; but LLAMA’s consistency in both retrieval-augmented and fine-tuned configurations confirms its general resilience.

Applied over models, RAG proved to be the most successful approach. Improvements in BLEU (e.g., LLAMA’s 0.163 vs. 0.025 for FT) and semantic metrics (e.g., BERTScore F1 for PHI: 0.868 for RAG vs. 0.850 for FT) consistently show it improving performance over standalone fine-tuning (FT). Sometimes the hybrid FT+RAG method beats RAG (e.g., PHI’s BLEU: 0.136), but RAG’s standalone efficacy and computational simplicity make it the recommended choice for balancing performance and practicality.

Though their gains are small relative to retrieval-augmented setups, GEMMA and MISTRAL provide competitive results for FT (e.g., GEMMA’s BLEU: 0.048; METEOR: 0.206). With LLAMA, RAG excels, scoring highest in BLEU (0.163) and ROUGE-L (0.407). For PHI, the FT+RAG hybrid is most successful since it achieves the highest BLEU (0.136) and BERTScore F1 (0.876), implying synergistic advantages from combining fine-tuning with retrieval.

LLAMA improves semantic coherence (SBERT Cosine Similarity: 0.822) and conventional measurements without adding hybrid complexity; thus it is best matched with RAG. PHI gains most from FT+RAG since it uses fine-tuning to increase retrieval gains—that is, BERTScore F1: 0.876. Although GEMMA and MISTRAL perform sufficiently with RAG (e.g., ROUGE-1: 0.386), their improvements over FT are less clear; hence RAG is a reasonable but modest choice. But QWEN shows little progress in the context of any approach, suggesting architectural changes or other approaches are needed.

### 6.6. Performance Comparison with Other Models

Throughout the studies reviewed, retrieval-augmented generation (RAG), fine-tuning (FT), and their hybrid approach (RAG+FT) exhibit different strengths based on the model architecture and task. The FT+RAG combination seems to produce the most dramatic performance improvements in various settings, particularly domain-specific applications like agriculture and Portuguese QA, where [10,15] saw additive accuracy gains. Fine-tuning alone also proves highly effective in structured settings like chatbot development, with [33] showing that it outperforms RAG and prompt engineering with the openassistant-guanaco model. By contrast, independent RAG was found to excel in situations of rapid adaptation to novel information or multisource data, particularly where high resource cost or catastrophic forgetting can hamper fine-tuning.

Yet, all such combinations are not effective in every case. Ref. [34] advises that fine-tuning can hinder RAG performance in real-world, multi-domain environments and advocates that integration methods need to be well-defined. In contrast, both [11] and an envisioned comparative study using LLAMA, GEMMA, MISTRAL, and QWEN models validates the flexibility and resilience of RAG as being the most efficient approach in tasks involving a great deal of knowledge. Table 6 shows the performance of fine-tuned models compared to other models, highlighting the gains brought about by adaptation. Generally, although FT is still useful for domain adaptation, RAG—and particularly RAG+FT—methods become general-purpose and effective methods for expanding LLM capabilities, especially in dynamic or low-resource settings.

### 6.7. Comparative Evaluation with Published Baselines

Our work was compared with the KnowTuning study [35], which utilized the same MedQuAD dataset and achieved a BERTScore of 0.842 and a METEOR score of 0.247 through model fine-tuning. In contrast, our best BERTScore was 0.891 using the FT approach on GEMMA, and our highest METEOR score was 0.206 using FT on MISTRAL. Although the METEOR score is slightly lower, it remains comparable. No prior work was found that applied RAG to the MedQuAD dataset.

One study [36] investigated FT, RAG, and FT+RAG methodologies on the Stanford QA dataset. They found a BLEU of 0.06 and METEOR of 0.44 for FT, BLEU of 0.06 and METEOR of 0.27 for RAG, and BLEU of 0.04 and METEOR of 0.16 for FT+RAG. Our best FT results were, in contrast, 0.05 BLEU and 0.20 METEOR on MISTRAL, 0.16 BLEU and 0.27 METEOR on RAG on LLAMA, and 0.15 BLEU and 0.26 METEOR on FT+RAG on LLAMA. Our models outperformed the RAG and FT+RAG approaches, but our fine-tuning results were slightly lower.

### 6.8. Carbon Emission

In the fine-tuning of five LLMs, the overall energy consumption and related carbon emissions were estimated. The overall energy usage for the total of five models was 2.688 kWh. For CO_2_ emissions, this activity emitted an estimated 1.162 kg of CO_2_. GEMMA used 0.589 kWh to produce 0.280 kg of CO_2_, whereas LLAMA needed 0.485 kWh, producing 0.194 kg of CO_2_. MISTRAL’s fine-tuning process took 0.51 kWh, emitting 0.242 kg of CO_2_, while PHI took 0.64 kWh, and its emissions resulted in 0.26 kg of CO_2_. Finally, QWEN’s fine-tuning process took 0.464 kWh, resulting in 0.186 kg of CO_2_ emissions. These results emphasize the energy and environmental effect of fine-tuning LLMs.

## 7. Limitations and Future Work

This section discusses the limitations of our current implementation, as well as problems with dataset coverage, evaluation size, and automated metric limitations. It also outlines potential avenues for future research.

### 7.1. Limitations

Several limitations should be noted, even though this study provides insightful information about tailoring large language models (LLMs) for answering medical questions. First, there is a chance of train-test leakage because some models might have been exposed to parts of MedQuAD during pre-training, even though it is considered an out-of-distribution dataset. Contamination checks should be incorporated into future work to guarantee dataset novelty. While the present study demonstrates the effectiveness of our approach on the MedQuAD medical QA dataset, the exclusive use of this single dataset necessarily limits the scope of our conclusions. Second, the models might not have fully optimized their learning because the fine-tuning was only carried out for one epoch, which could limit performance gains. Third, the assessment makes extensive use of automatic metrics like BERTScore and BLEU, which may not precisely represent clinical correctness because they deviate from expert opinions. Finally, metric variance may rise as a result of the comparatively small evaluation set. Larger and more varied clinical benchmarks are necessary for drawing solid conclusions, even though bootstrapping was used to address this.

### 7.2. Future Work

Although fine-tuning, RAG, and their combination (FT+RAG) across several quantized LLMs show encouraging results, several paths remain open for future research. First, experimenting with larger batch sizes and extending the training length beyond one epoch could help to improve convergence and model generalization. Particularly for models like GEMMA and MISTRAL, which showed inconsistent gains with RAG, investigating alternative prompt structures and fine-tuning templates may help to improve performance.

As seen with GEMMA and QWEN during SBERT-based evaluation, another difficulty is the possibility of semantic drift brought about by retrieval in some architectures. Future research could look at hybrid re-ranking methods or more strong retrieval filters to reduce noise and improve the relevance of obtained papers. Furthermore, although 4-bit quantization provided significant memory savings, it occasionally resulted in performance degradation—particularly for models less-suited to low-precision computation. More precise quantization-aware training and post-training optimization techniques might assist to solve this problem.

Firstly, for domain-specific tasks like clinical decision support, scalability of the present architecture in real-time applications remains an open challenge as well. Secondly, future investigations employing alternative question-answering datasets would help establish the robustness and domain-transfer capabilities of the methodology. Integrating structured medical knowledge graphs, enhancing long-context support, and optimizing the vector database (Chromadb) indexing technique could help to improve retrieval relevance and model responsiveness even more. Finally, including real-world clinical benchmarks and human expert validation into evaluation procedures will help to give a more complete assessment of the dependability and safety of the model [37].

## 8. Conclusions

This research conducted a thorough comparison of five quantized large language models—Meta-Llama-3.1-8B, Phi-3.5-mini-instruct, Gemma-2-9B, Mistral-7B-Instruct, and Qwen2.5-7B—on three adaptation strategies: fine-tuning (FT), retrieval-augmented generation (RAG), and their combination (FT+RAG). Each model was fine-tuned on a curated medical QA dataset using 4-bit quantization for memory savings. Evaluation was conducted using a rich variety of metrics, such as perplexity, BLEU, GLEU, ROUGE-1, ROUGE-2, ROUGE-L, METEOR, Precision, Recall, F1, BERTScore Precision, BERTScore Recall, BERTScore F1, SBERT Cosine Similarity, and Negation-Aware Semantic Similarity.

The comparison of perplexity scores before and after fine-tuning shows essential information regarding model performance and flexibility. Some architectures showed tremendous gains in perplexity, pointing to high compatibility with the domain-specific fine-tuning method. However, other models showed rising perplexity scores, implying some level of deficiency in their flexibility to adjust to the target data distribution. These results coincide with larger evaluation metrics from the study, whereby models that are best at reducing perplexity also performed well across semantic coherence and task-specific measurements such as BERTScore and ROUGE.

The results show that LLAMA shows consistency in both conventional and semantic evaluation measures, proving its resilience in text generating tasks. Especially the RAG approach proved to be the most efficient method generally, greatly improving model performance by using outside knowledge retrieval. For some models, such as PHI, the hybrid FT+RAG approach proved especially helpful, implying that in particular situations combining fine-tuning with retrieval can have synergistic effects. The *p*-values and intervals demonstrated that RAG and FT+RAG performed significantly better than fine-tuning alone in few of the models, with PHI benefiting more from the hybrid approach and LLAMA demonstrating improvements in all metrics. These results show the need for choosing the appropriate model and technique depending on computational restrictions and job requirements.

Moreover, the analysis emphasizes how feasible 4-bit quantized models are in balancing efficiency and performance, which makes them sensible for actual uses with limited means. Although most models gained from retrieval augmentation, the degree of improvement differed; QWEN showed little changes, suggesting possible architectural constraints or the necessity of other optimization techniques. Higher-precision quantization or dynamic retrieval methods could be investigated in future work to improve these models even more. All things considered, this study provides insightful analysis for practitioners hoping to use effective, high-performance language models in settings with limited resources.

## Figures and Tables

**Table 1:** Related research on Fine Tuning vs. RAG.

Author(s)	Title	Method(s)	Key Takeaways
[11]	Fine-Tuning or Retrieval? Comparing Knowledge Injection in LLMs	FT, RAG	RAG was more effective at injecting both novel and known knowledge.
[12]	Fine Tuning vs. Retrieval Augmented Generation for Less Popular Knowledge	FT, RAG	RAG outperformed FT for rare knowledge; FT improved with data augmentation.
[13]	A Comparative Study of DSL Code Generation: Fine-Tuning vs. Optimized Retrieval Augmentation	FT, RAG	RAG performed better for unseen APIs; FT had higher code similarity.
[14]	Systematic Analysis of Retrieval-Augmented Generation-Based Medical Chatbots	FT, RAG, FT+RAG	FT+RAG achieved best results for medical QA under resource constraints.
[15]	Adapting LLMs to New Domains: A Comparative Study of Fine-Tuning and RAG Strategies for Portuguese QA Tasks	FT, RAG, FT+RAG	Hybrid FT+RAG approach provided best domain adaptation for Portuguese QA.
[16]	Investigating the Performance of Retrieval- Augmented Generation and Fine-Tuning for the Development of AI-Driven Knowledge-Based Systems	FT, RAG	RAG showed higher BLEU and ROUGE; FT had slightly better creativity.
[17]	Towards Understanding Systems Trade-offs in Retrieval-Augmented Generation Model Inference	RAG	RAG offers no retraining needs but incurs latency and memory costs.
[18]	Establishing Performance Baselines in Fine-Tuning, Retrieval-Augmented Generation and Soft-Prompting for Non-Specialist LLM Users	FT, RAG, Soft Prompting	RAG had best accuracy for post-2021 queries; soft prompting improved outcomes.
[19]	Development and Testing of Retrieval Augmented Generation in Large Language Models	RAG, FT	GPT-4 with RAG achieved 91.4% accuracy, surpassing human experts.
[20]	Retrieval Augmented Generation (RAG) and Beyond: A Comprehensive Survey on How to Make your LLMs use External Data More Wisely	FT, RAG, Hybrid	Survey emphasizes hybrid strategies to meet complex real-world LLM tasks.

**Table 2:** Perplexity scores before and after MedQuAD fine-tuning across the five models. ↓ indicate increasing order.

Model	Original (Unsloth) PPL (↓)	Fine Tuned (MedQuAD) PPL (↓)	ΔPPL
PHI	34.79 ± 0.8	33.64 ± 0.7	−1.15
QWEN	38.15 ± 1.2	41.15 ± 1.5	+3.00
LLAMA	51.52 ± 2.1	48.48 ± 1.9	−3.04
MISTRAL	39.73 ± 1.4	47.84 ± 2.0	+8.11
GEMMA	186.43 ± 5.7	86.79 ± 3.2	−99.64

**Table 3:** Evaluation metrics for all models across the FT, RAG, and FT+RAG methods.

Model	Method	BLEU	GLEU	R-1	R-2	R-L	METEOR	Precision	Recall	F1	BS_P	BS_R	BS_F1	SB_CS	NASS
GEMMA	FT	0.048	0.129	0.313	0.118	0.296	0.196	0.569	0.336	0.392	0.888	0.848	0.867	0.802	0.857
	RAG	0.123	0.190	0.354	0.212	0.342	0.235	0.585	0.357	0.391	0.881	0.849	0.864	0.760	0.819
	FT+RAG	0.104	0.181	0.351	0.193	0.338	0.240	0.586	0.367	0.405	0.890	0.854	0.871	0.791	0.841
PHI	FT	0.036	0.122	0.267	0.076	0.247	0.200	0.433	0.387	0.390	0.855	0.846	0.850	0.781	0.824
	RAG	0.111	0.190	0.366	0.212	0.354	0.243	0.602	0.367	0.417	0.891	0.846	0.868	0.810	0.875
	FT+RAG	0.136	0.217	0.391	0.250	0.380	0.258	0.651	0.366	0.435	0.903	0.852	0.876	0.815	0.881
QWEN	FT	0.038	0.109	0.276	0.106	0.263	0.162	0.610	0.265	0.334	0.892	0.838	0.864	0.788	0.856
	RAG	0.048	0.128	0.306	0.168	0.294	0.161	0.720	0.215	0.304	0.908	0.835	0.869	0.773	0.837
	FT+RAG	0.042	0.130	0.302	0.166	0.290	0.160	0.716	0.232	0.316	0.911	0.838	0.872	0.787	0.859
LLAMA	FT	0.025	0.089	0.262	0.080	0.245	0.146	0.571	0.248	0.315	0.881	0.834	0.857	0.766	0.816
	RAG	0.163	0.237	0.418	0.280	0.407	0.279	0.711	0.369	0.436	0.916	0.862	0.888	0.822	0.898
	FT+RAG	0.153	0.236	0.423	0.299	0.413	0.268	0.756	0.342	0.419	0.925	0.860	0.891	0.819	0.908
MISTRAL	FT	0.055	0.136	0.315	0.116	0.297	0.206	0.527	0.355	0.401	0.879	0.851	0.864	0.803	0.845
	RAG	0.093	0.186	0.386	0.235	0.368	0.220	0.762	0.290	0.389	0.918	0.850	0.883	0.821	0.878
	FT+RAG	0.089	0.172	0.346	0.205	0.331	0.207	0.694	0.291	0.370	0.907	0.846	0.875	0.796	0.857

**Table 4:** The table shows 95% confidence intervals for evaluation metrics (shown only where *p* < 0.05 in Table 5). Non-significant metrics are marked as “—”.

Model	Method	BLEU	GLEU	R-1	R-2	R-L	METEOR	Precision	Recall	F1	BS_P	BS_R	BS_F1	SB_CS	NASS
GEMMA	FT	(0.039, 0.058)	(0.118, 0.140)	—	(0.107, 0.130)	—	—	—	—	—	—	—	—	(0.784, 0.820)	—
	RAG	(0.088, 0.165)	(0.155, 0.226)	—	(0.166, 0.258)	—	—	—	—	—	—	—	—	(0.730, 0.789)	—
	FT+RAG	—	(0.151, 0.214)	—	—	—	—	—	—	—	—	—	—	(0.766, 0.816)	—
PHI	FT	(0.028, 0.045)	(0.113, 0.131)	(0.253, 0.280)	(0.067, 0.085)	(0.234, 0.262)	—	(0.408, 0.458)	—	(0.374, 0.405)	(0.851, 0.859)	—	(0.845, 0.855)	(0.760, 0.801)	(0.803, 0.842)
	RAG	(0.084, 0.142)	(0.164, 0.219)	(0.332, 0.399)	(0.176, 0.253)	(0.320, 0.389)	—	(0.560, 0.644)	—	—	(0.884, 0.899)	(0.839, 0.853)	(0.862, 0.874)	—	(0.844, 0.900)
	FT+RAG	(0.103, 0.172)	(0.186, 0.252)	(0.350, 0.433)	(0.201, 0.295)	(0.338, 0.422)	—	(0.606, 0.696)	—	(0.402, 0.469)	(0.893, 0.911)	(0.844, 0.860)	(0.869, 0.885)	(0.794, 0.835)	(0.854, 0.911)
QWEN	FT	—	—	—	(0.093, 0.117)	—	—	(0.580, 0.641)	(0.234, 0.296)	—	(0.887, 0.897)	—	(0.859, 0.869)	—	—
	RAG	—	—	—	(0.129, 0.204)	—	—	(0.676, 0.767)	(0.186, 0.248)	—	(0.900, 0.917)	—	—	—	—
	FT+RAG	—	—	—	(0.135, 0.199)	—	—	(0.669, 0.762)	—	—	(0.902, 0.921)	—	(0.867, 0.880)	—	—
LLAMA	FT	(0.019, 0.033)	(0.080, 0.100)	(0.246, 0.277)	(0.071, 0.091)	(0.231, 0.260)	(0.129, 0.163)	(0.542, 0.600)	(0.220, 0.274)	(0.293, 0.336)	(0.875, 0.886)	(0.828, 0.841)	(0.852, 0.862)	(0.747, 0.787)	(0.794, 0.835)
	RAG	(0.120, 0.204)	(0.197, 0.279)	(0.372, 0.463)	(0.233, 0.329)	(0.363, 0.448)	(0.236, 0.323)	(0.669, 0.751)	(0.319, 0.418)	(0.392, 0.479)	(0.907, 0.926)	(0.852, 0.873)	(0.878, 0.896)	(0.797, 0.843)	(0.873, 0.922)
	FT+RAG	(0.113, 0.194)	(0.196, 0.278)	(0.381, 0.465)	(0.249, 0.347)	(0.370, 0.457)	(0.226, 0.309)	(0.708, 0.803)	(0.297, 0.390)	(0.379, 0.463)	(0.915, 0.936)	(0.850, 0.870)	(0.882, 0.899)	(0.795, 0.840)	(0.884, 0.932)
MISTRAL	FT	—	—	(0.298, 0.332)	(0.105, 0.128)	(0.282, 0.313)	—	(0.496, 0.557)	(0.329, 0.381)	—	(0.873, 0.885)	—	(0.859, 0.869)	(0.787, 0.818)	(0.829, 0.861)
	RAG	—	—	(0.350, 0.424)	(0.194, 0.271)	(0.332, 0.408)	—	(0.720, 0.802)	(0.254, 0.329)	—	(0.910, 0.928)	—	(0.875, 0.891)	(0.805, 0.839)	(0.852, 0.901)
	FT+RAG	—	—	—	(0.169, 0.245)	—	—	(0.649, 0.739)	(0.253, 0.328)	—	(0.898, 0.917)	—	(0.868, 0.883)	—	—

Note: CIs are shown for methods where at least one pairwise comparison (FT vs. RAG, FT vs. FT+RAG, or RAG vs. FT+RAG) had *p* < 0.05 in Table 5.

**Table 5:** Statistical significance (*p*-values) for comparisons between FT, RAG, and FT+RAG methods.

Model	Comparison	BLEU	GLEU	R-1	R-2	R-L	METEOR	Precision	Recall	F1	BS_P	BS_R	BS_F1	SB_CS	NASS
GEMMA	FT vs. RAG	0.042	0.037	0.229	0.006	0.176	0.419	0.883	0.644	0.519	0.289	0.688	0.513	0.007	0.229
	FT vs. FT+RAG	0.235	0.034	0.345	0.190	0.316	0.089	0.891	0.210	0.601	0.916	0.235	0.669	0.194	0.945
	RAG vs. FT+RAG	0.941	0.677	0.665	0.720	0.724	0.438	0.958	0.496	0.329	0.163	0.170	0.078	0.022	0.245
PHI	FT vs. RAG	3.4 × $10^{-5}$	3.6 × $10^{-5}$	2.3 × $10^{-6}$	1.6 × $10^{-8}$	8.6 × $10^{-7}$	0.028	1.3 × $10^{-10}$	0.150	0.154	3.9 × $10^{-10}$	0.296	3.2 × $10^{-5}$	0.050	2.6 × $10^{-4}$
	FT vs. FT+RAG	1.4 × $10^{-7}$	6.3 × $10^{-6}$	1.0 × $10^{-6}$	2.0 × $10^{-9}$	2.6 × $10^{-7}$	0.004	1.4 × $10^{-11}$	0.133	0.039	2.3 × $10^{-11}$	0.217	9.9 × $10^{-8}$	0.014	3.0 × $10^{-5}$
	RAG vs. FT+RAG	0.289	0.043	0.170	0.054	0.139	0.284	0.088	0.985	0.203	0.009	0.005	0.002	0.598	0.122
QWEN	FT vs. RAG	0.891	0.477	0.233	0.044	0.345	0.751	1.3 × $10^{-4}$	0.026	0.207	0.005	0.306	0.296	0.135	0.983
	FT vs. FT+RAG	0.523	0.170	0.196	0.003	0.231	0.404	4.2 × $10^{-4}$	0.072	0.205	9.5 × $10^{-4}$	0.572	0.035	0.688	0.434
	RAG vs. FT+RAG	0.672	0.414	0.893	0.832	0.924	0.559	0.966	0.280	0.347	0.452	0.268	0.275	0.220	0.186
LLAMA	FT vs. RAG	1.2 × $10^{-7}$	2.2 × $10^{-8}$	8.6 × $10^{-9}$	4.2 × $10^{-9}$	8.4 × $10^{-9}$	2.0 × $10^{-6}$	2.0 × $10^{-7}$	9.5 × $10^{-5}$	7.5 × $10^{-6}$	2.2 × $10^{-8}$	5.2 × $10^{-6}$	3.5 × $10^{-8}$	1.8 × $10^{-5}$	2.0 × $10^{-5}$
	FT vs. FT+RAG	2.9 × $10^{-6}$	2.2 × $10^{-7}$	7.2 × $10^{-8}$	1.0 × $10^{-9}$	6.6 × $10^{-8}$	2.1 × $10^{-5}$	5.6 × $10^{-9}$	3.4 × $10^{-3}$	3.2 × $10^{-4}$	1.6 × $10^{-10}$	3.8 × $10^{-5}$	5.9 × $10^{-9}$	1.0 × $10^{-4}$	6.7 × $10^{-7}$
	RAG vs. FT+RAG	0.512	0.748	0.657	0.117	0.591	0.141	0.069	0.117	0.336	0.052	0.482	0.097	0.846	0.167
MISTRAL	FT vs. RAG	0.513	0.053	0.004	9.2 × $10^{-6}$	0.004	0.788	1.2 × $10^{-11}$	3.7 × $10^{-4}$	0.387	2.9 × $10^{-9}$	0.587	6.8 × $10^{-5}$	0.037	1.7 × $10^{-3}$
	FT vs. FT+RAG	0.513	0.537	0.256	0.004	0.245	0.384	3.9 × $10^{-8}$	0.001	0.051	5.1 × $10^{-6}$	0.099	0.026	0.825	0.321
	RAG vs. FT+RAG	0.963	0.686	0.181	0.252	0.195	0.867	0.001	0.781	0.650	0.005	0.765	0.049	0.268	0.091

**Table 6:** Most effective method comparison of proposed models with other models.

Paper	Models Used	Most Effective Method	Notes
[10]	Llama2-13B, GPT-3.5, GPT-4	FT+RAG	Fine-tuning improved accuracy by 6 percentage points and RAG added 5 more percentage points
[11]	Not explicitly named, generic LLMs	RAG	RAG consistently outperformed fine-tuning for both new and existing knowledge
[15]	Not specified	FT+RAG	Combined approach yielded best results in Portuguese QA tasks
[33]	openassistant-guanaco	FT	Achieved highest accuracy (87.8%) and BLEU (0.81); RAG and prompt engineering were competitive
[34]	Not specified	RAG	Fine-tuning degraded RAG performance across domains
Proposed	Llama-3.1-8B, Gemma-2-9B, Mistral-7B-Instruct, Qwen2.5-7B, Phi-3.5-Mini-Instruct	RAG	retrieval-augmented generation (RAG) emerged as most effective adaptation strategy.

## Data Availability

Data and code presented in this study have been openly shared on GitHub by the authors at https://github.com/bhagyajit6/FTvsRAG.git (accessed on 19 June 2025) and Supplementary Materials.

## Supplementary Materials

The following supporting information can be downloaded at: https://www.mdpi.com/article/10.3390/bioengineering12070687/s1, Supplementary Material Figure S1: Model-wise GLEU score comparison: fine-tuning, RAG, and FT+RAG strategies. Supplementary Material Figure S2: Model-wise METEOR score comparison: fine-tuning, RAG, and FT+RAG strategies. Supplementary Material Figure S3: Model-wise ROUGE-1 score comparison: fine-tuning, RAG, and FT+RAG strategies. Supplementary Material Figure S4: Model-wise ROUGE-2 score comparison: fine-tuning, RAG, and FT+RAG strategies. Supplementary Material Figure S5: Model-wise ROUGE-L score comparison: fine-tuning, RAG, and FT+RAG strategies. Supplementary Material Figure S6: Model-wise precision comparison: fine-tuning, RAG, and FT+RAG strategies. Supplementary Material Figure S7: Model-wise recall comparison: fine-tuning, RAG, and FT+RAG strategies. Supplementary Material Figure S8: Model-wise F1 score comparison: fine-tuning, RAG, and FT+RAG strategies. Supplementary Material Figure S9: Model-wise BERTScore Precision comparison: fine-tuning, RAG, and FT+RAG strategies. Supplementary Material Figure S10: Model-wise BERTScore Recall comparison: Fine-Tuning, RAG, and FT+RAG strategies. Supplementary Material Figure S11: Model-wise SBERT Cosine Similarity comparison: fine-tuning, RAG, and FT+RAG strategies. Supplementary Material Figure S12: Model-wise Negation-Aware Semantic Similarity comparison: fine-tuning, RAG, and FT+RAG strategies.

## Abbreviations

The following abbreviations are used in this manuscript:

LLM	Large Language Model
GEMMA	Gemma-2-9B
LLAMA	Llama-3.1-8B
QWEN	Qwen2.5-7B
MISTRAL	Mistral-7B-Instruct
PHI	Phi-3.5-mini-instruct
FT	Fine-Tuning
RAG	Retrieval-Augmented Generation
FT+RAG	Fine-Tuning & Retrieval-Augmented Generation
LoRA	Low-Rank Adaptation
QLoRA	Quantized Low-Rank Adaptation
PPL	Perplexity
ROUGE	Recall-Oriented Understudy for Gisting Evaluation
BLEU	Bilingual Evaluation Understudy
GLEU	Google-Bilingual Evaluation Understudy
R-1	ROUGE-1
R-2	ROUGE-2
R-L	ROUGE-L
BERT	Bidirectional Encoder Representations from Transformers
SBERT	Sentence-BERT
BS_P	BERTScore Precision
BS_R	BERTScore Recall
BS_F1	BERTScore F1
SB_CS	SBERT Cosine Similarity
NASS	Negation Aware Semantic Similarity
