# Supplementary material for: Medical LLMs: Fine-Tuning vs. Retrieval-Augmented Generation
Source: Bioengineering (Basel). 2025 Jun 24;12(7):687. doi: 10.3390/bioengineering12070687 (PMC12292519; doi:10.3390/bioengineering12070687)
Supplement: Supplementary file 1 [file bioengineering-12-00687-s001.zip › bioengineering-3689564-supplementary.pdf]

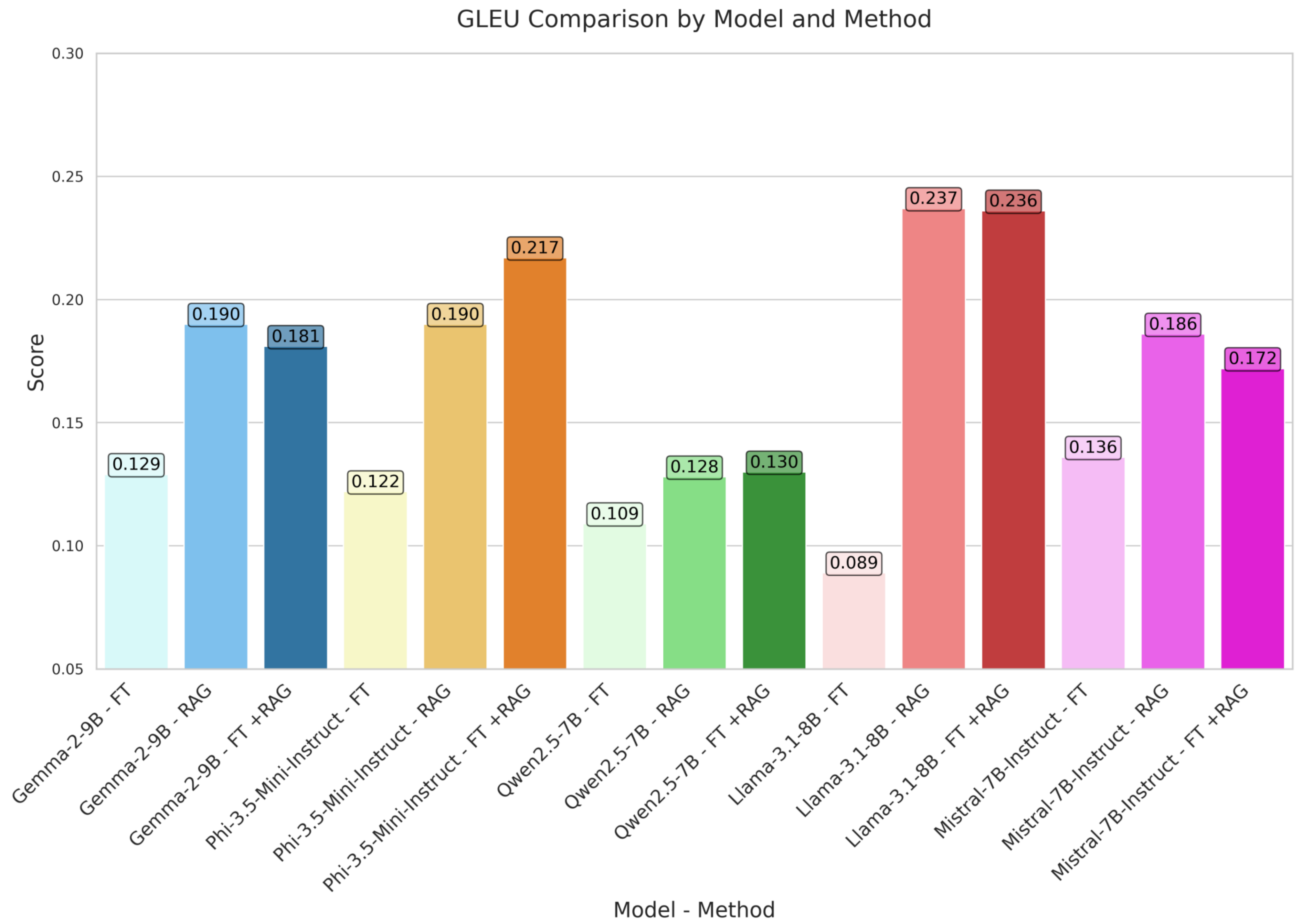

Figure S1: Model-wise GLEU Score Comparison: Fine-Tuning, RAG, and FT+RAG Strategies.

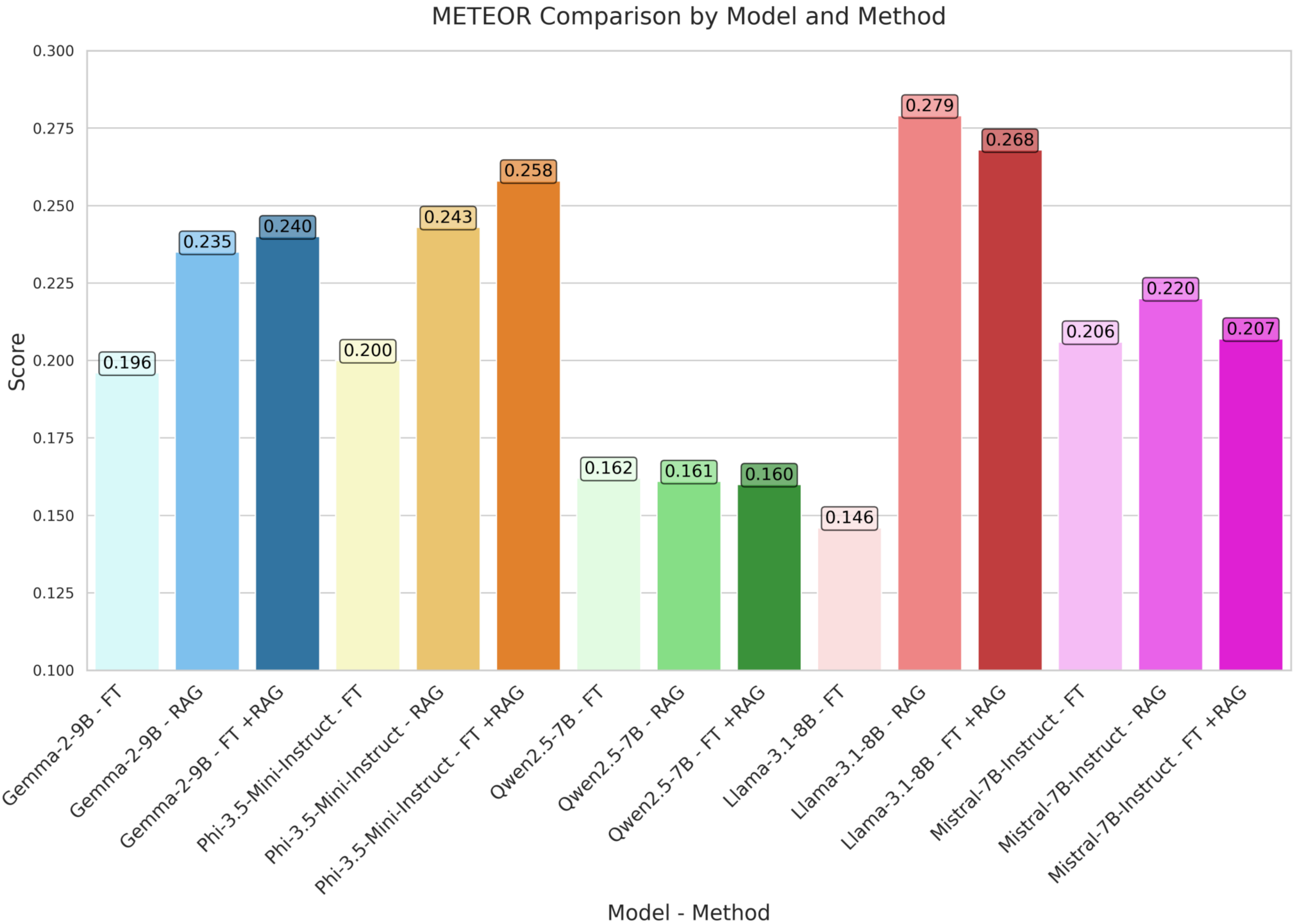

Figure S2: Model-wise METEOR Score Comparison: Fine-Tuning, RAG, and FT+RAG Strategies.

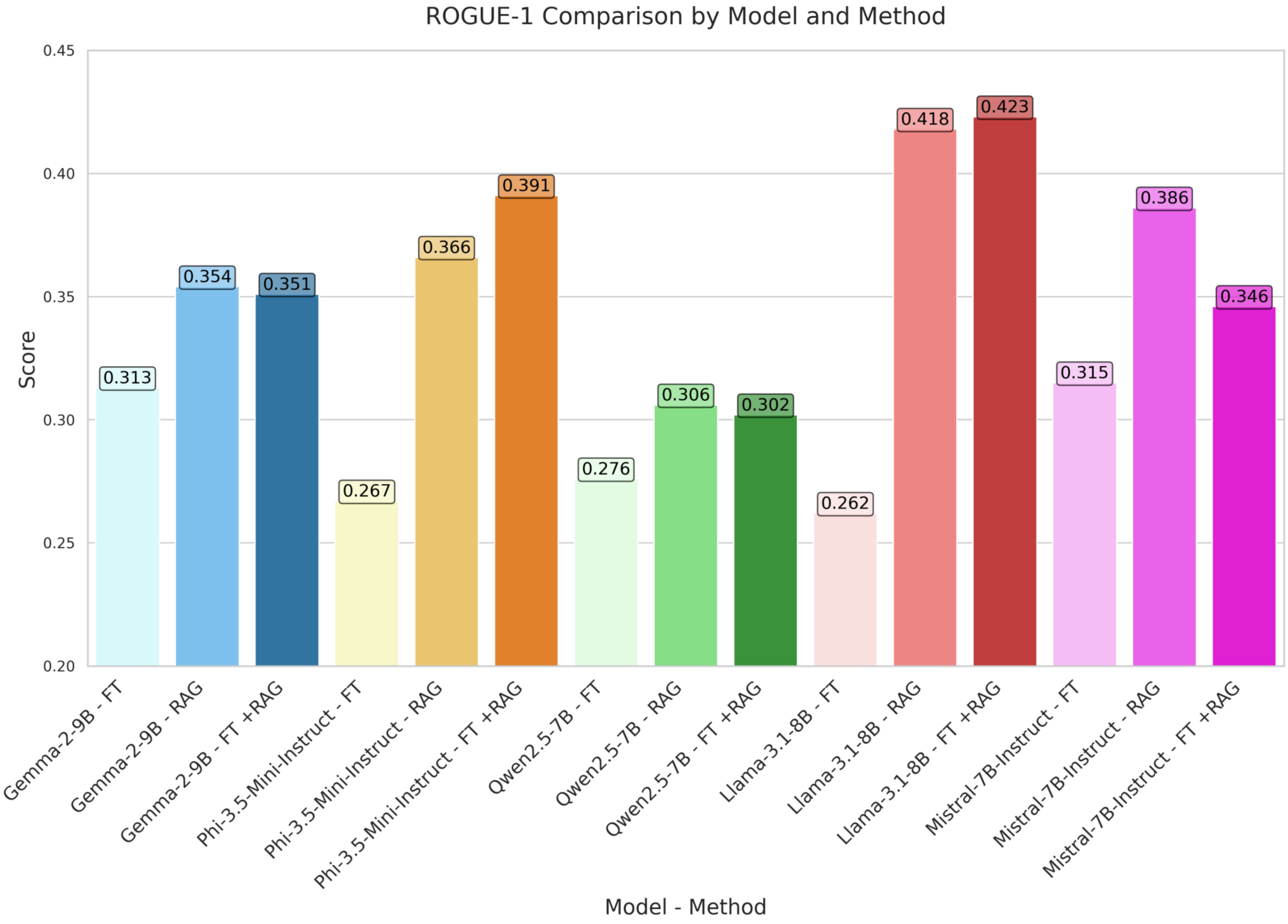

Figure S3: Model-wise ROUGE-1 Score Comparison: Fine-Tuning, RAG, and FT+RAG Strategies.

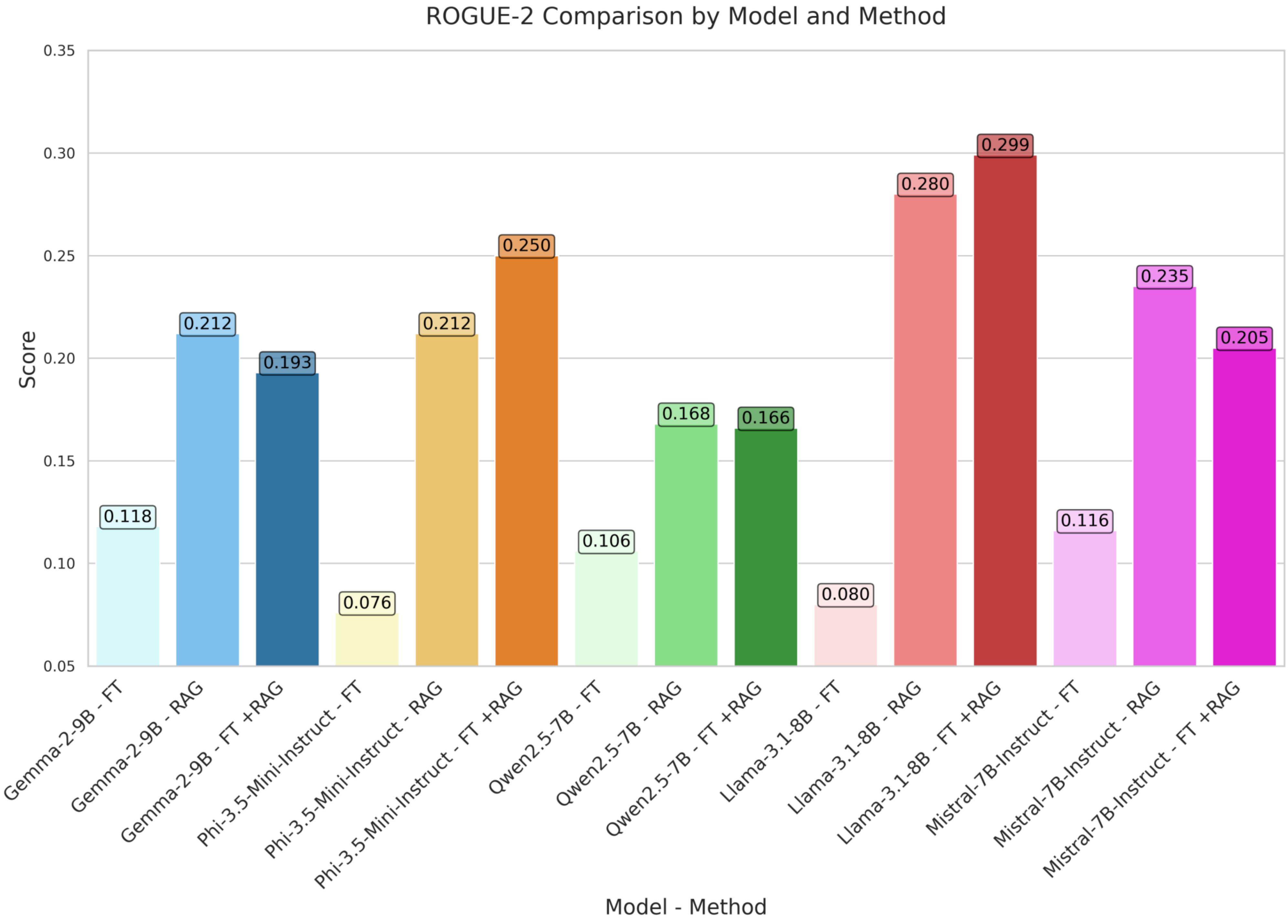

Figure S4: Model-wise ROUGE-2 Score Comparison: Fine-Tuning, RAG, and FT+RAG Strategies.

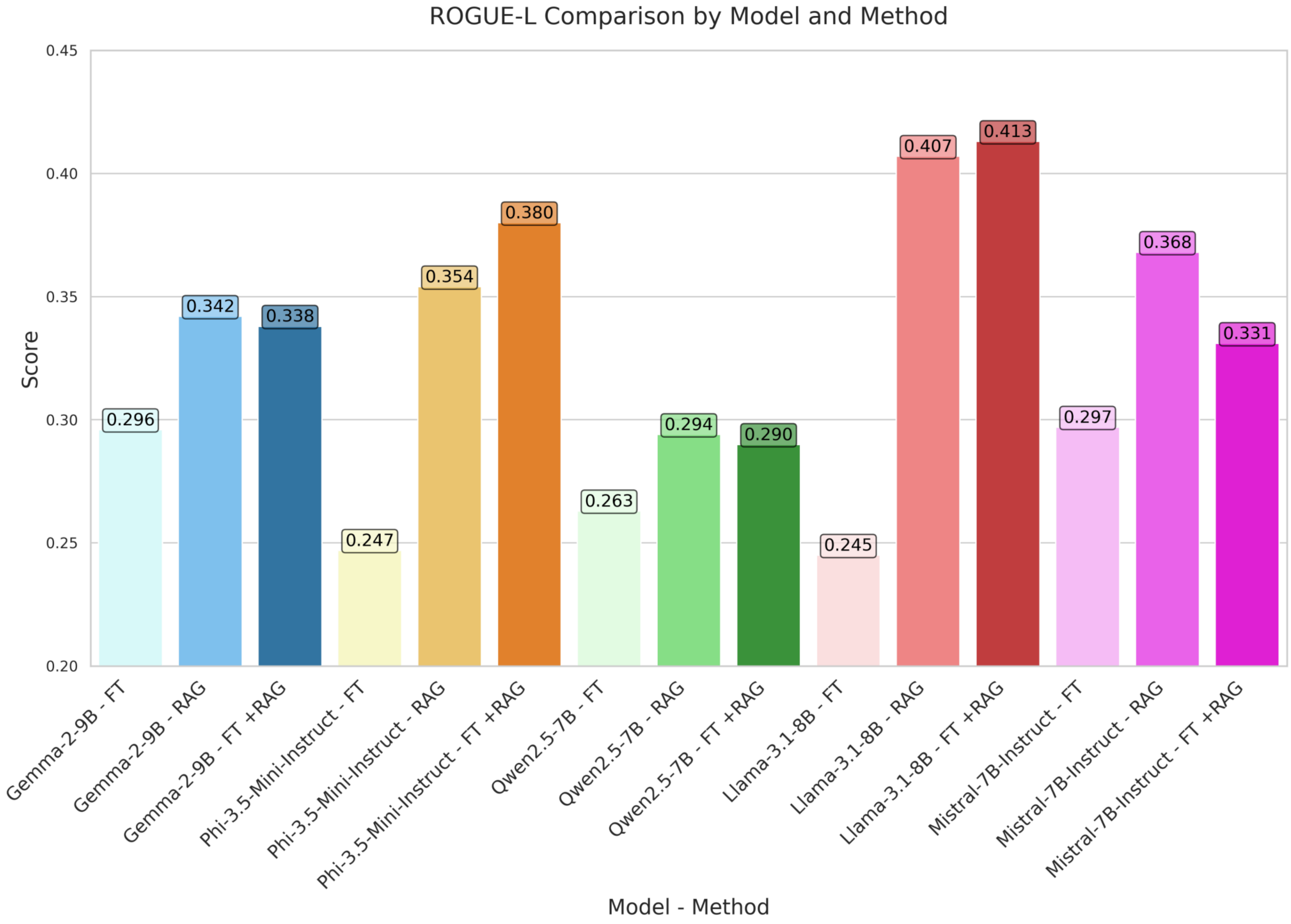

Figure S5: Model-wise ROUGE-L Score Comparison: Fine-Tuning, RAG, and FT+RAG Strategies.

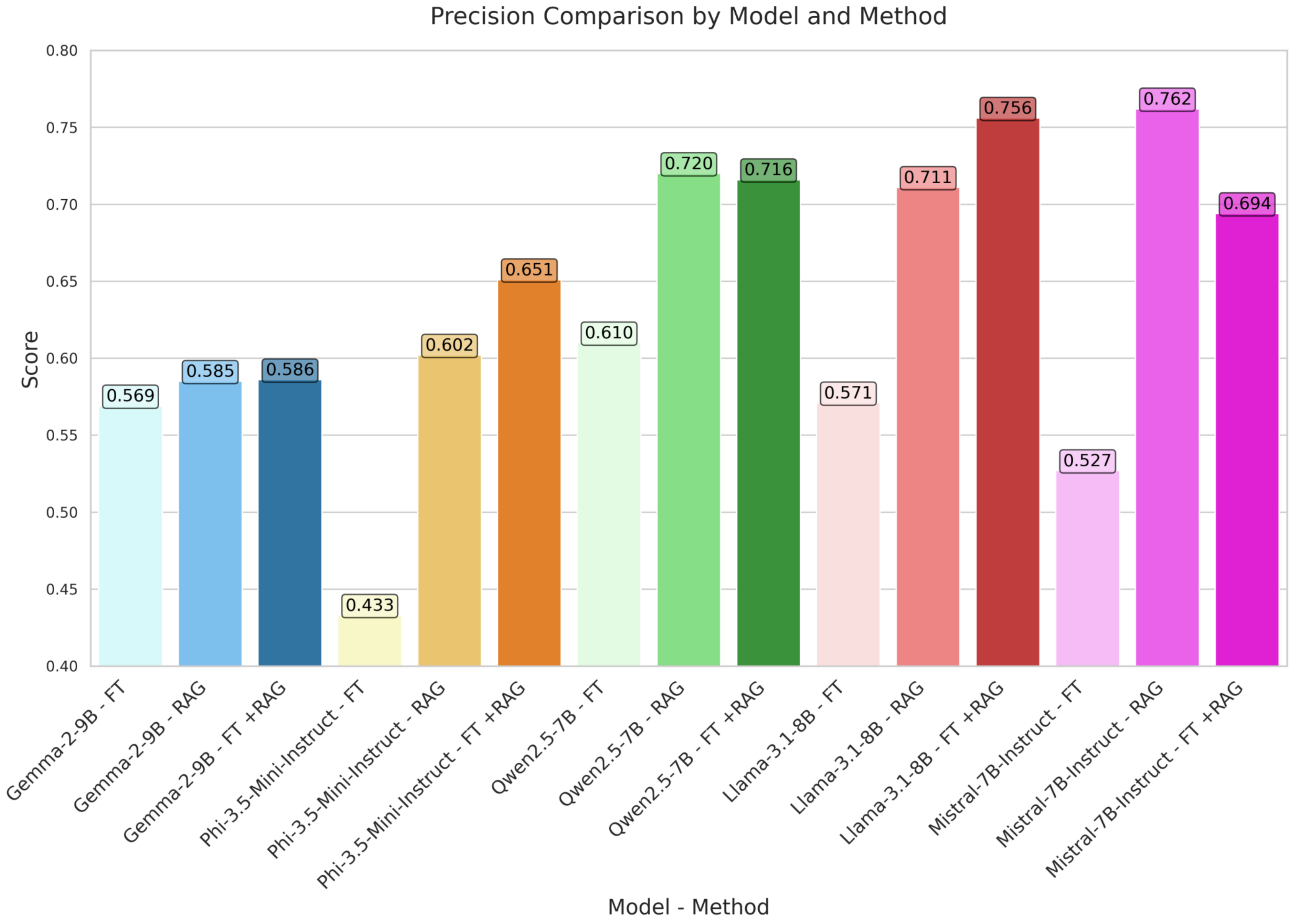

Figure S6: Model-wise Precision Comparison: Fine-Tuning, RAG, and FT+RAG Strategies.

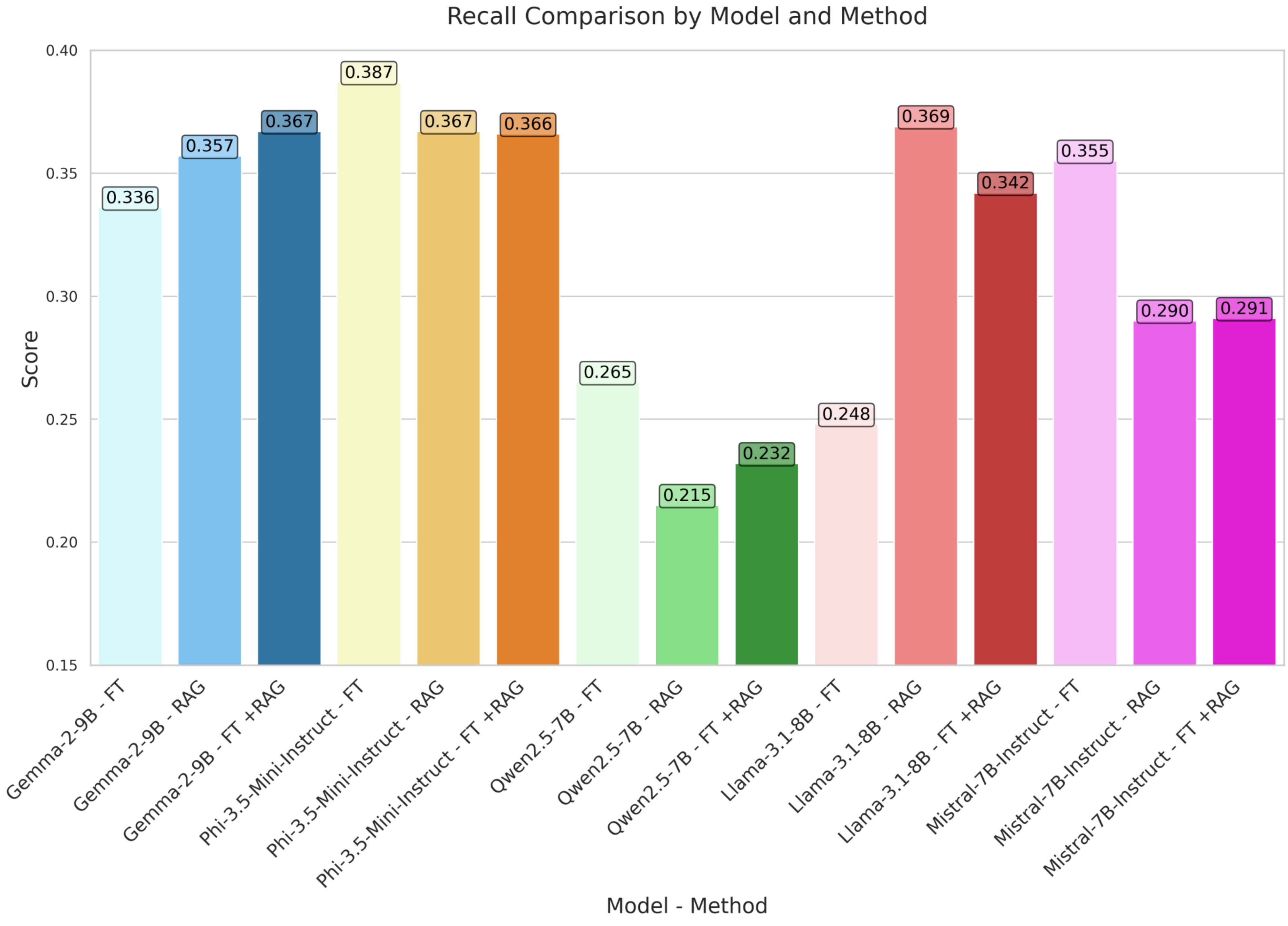

Figure S7: Model-wise Recall Comparison: Fine-Tuning, RAG, and FT+RAG Strategies.

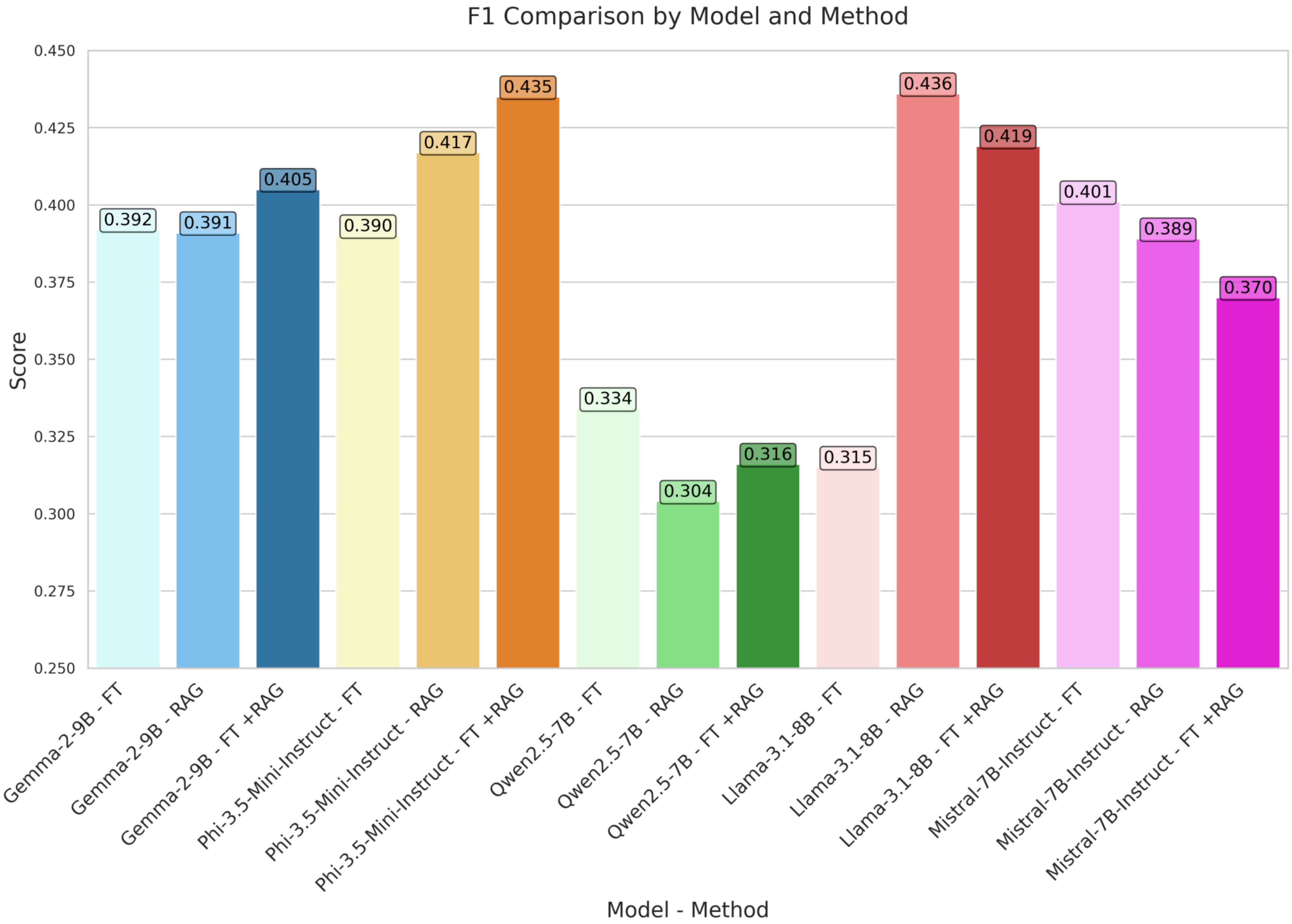

Figure S8: Model-wise F1 Score Comparison: Fine-Tuning, RAG, and FT+RAG Strategies.

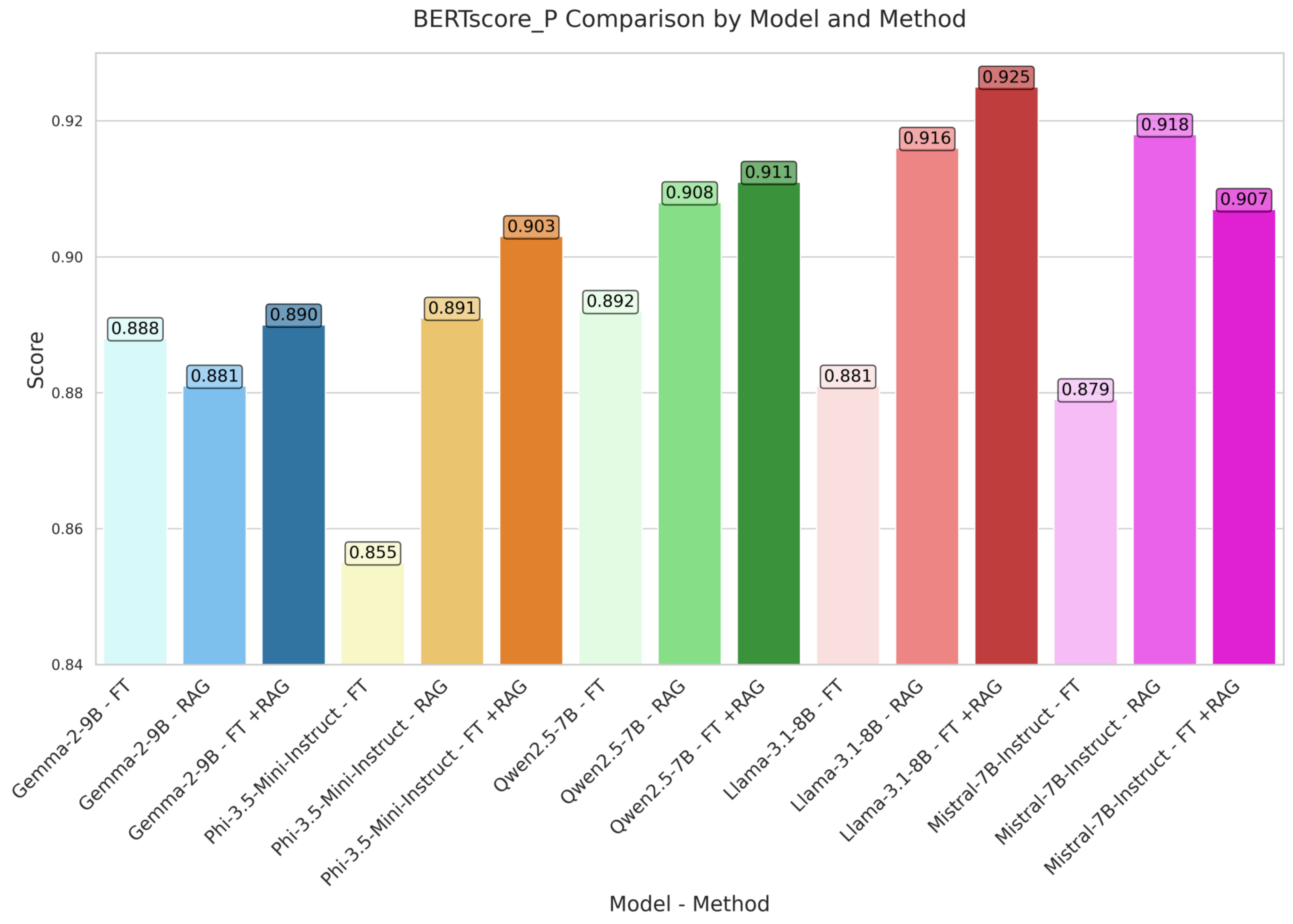

Figure S9: Model-wise BERTScore Precision Comparison: Fine-Tuning, RAG, and FT+RAG Strategies.

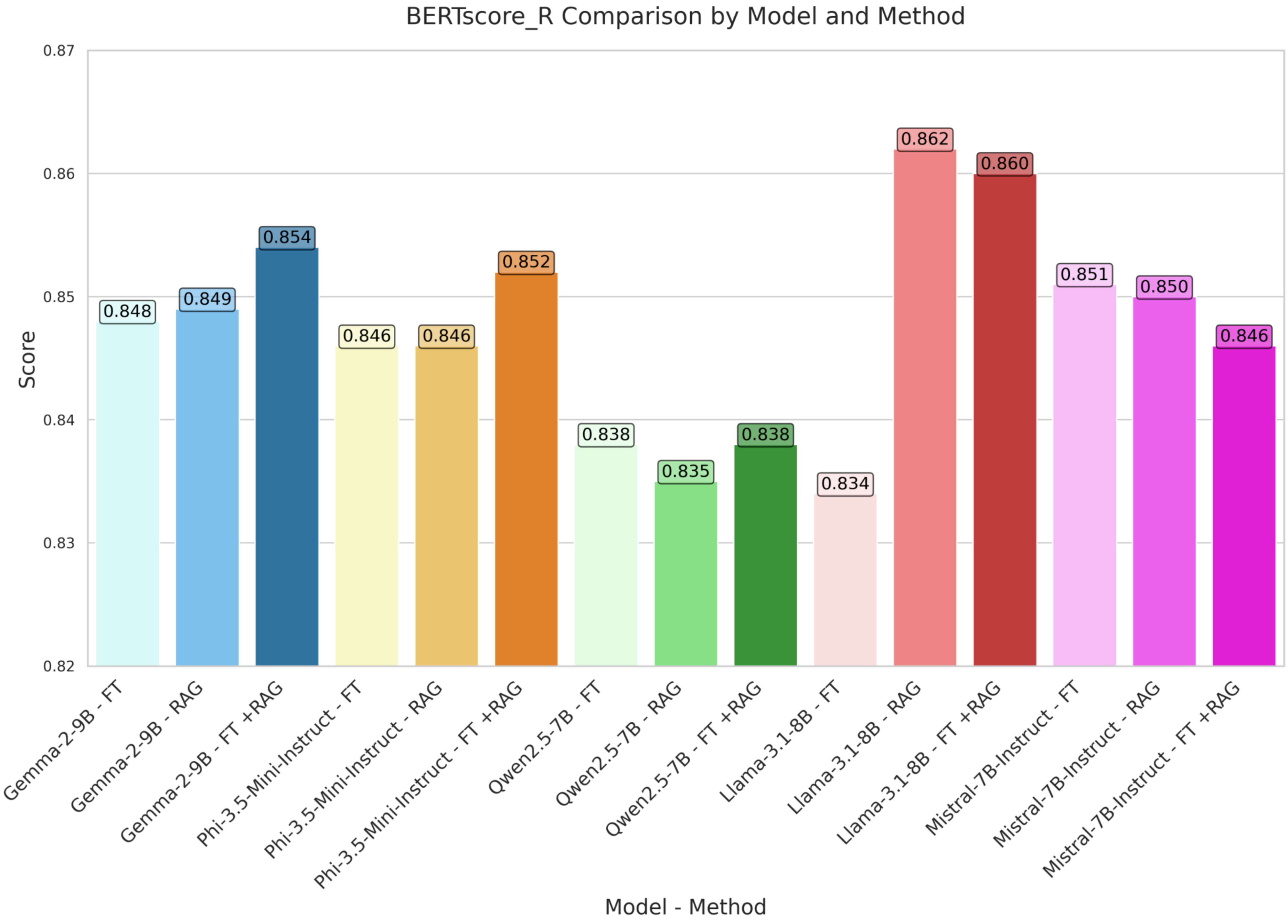

Figure S10: Model-wise BERTScore Recall Comparison: Fine-Tuning, RAG, and FT+RAG Strategies.

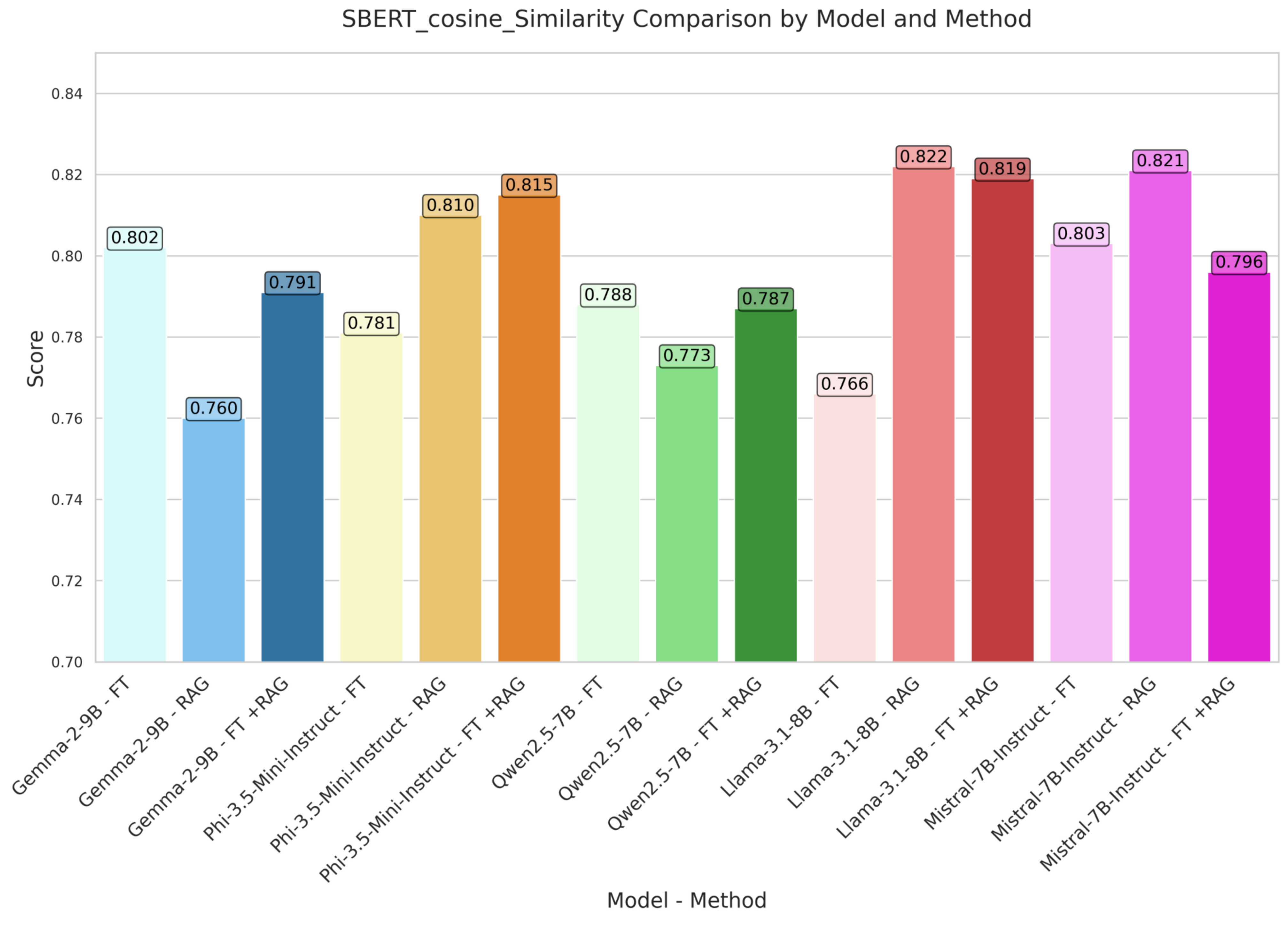

Figure S11: Model-wise SBERT Cosine Similarity Comparison: Fine-Tuning, RAG, and FT+RAG Strategies.

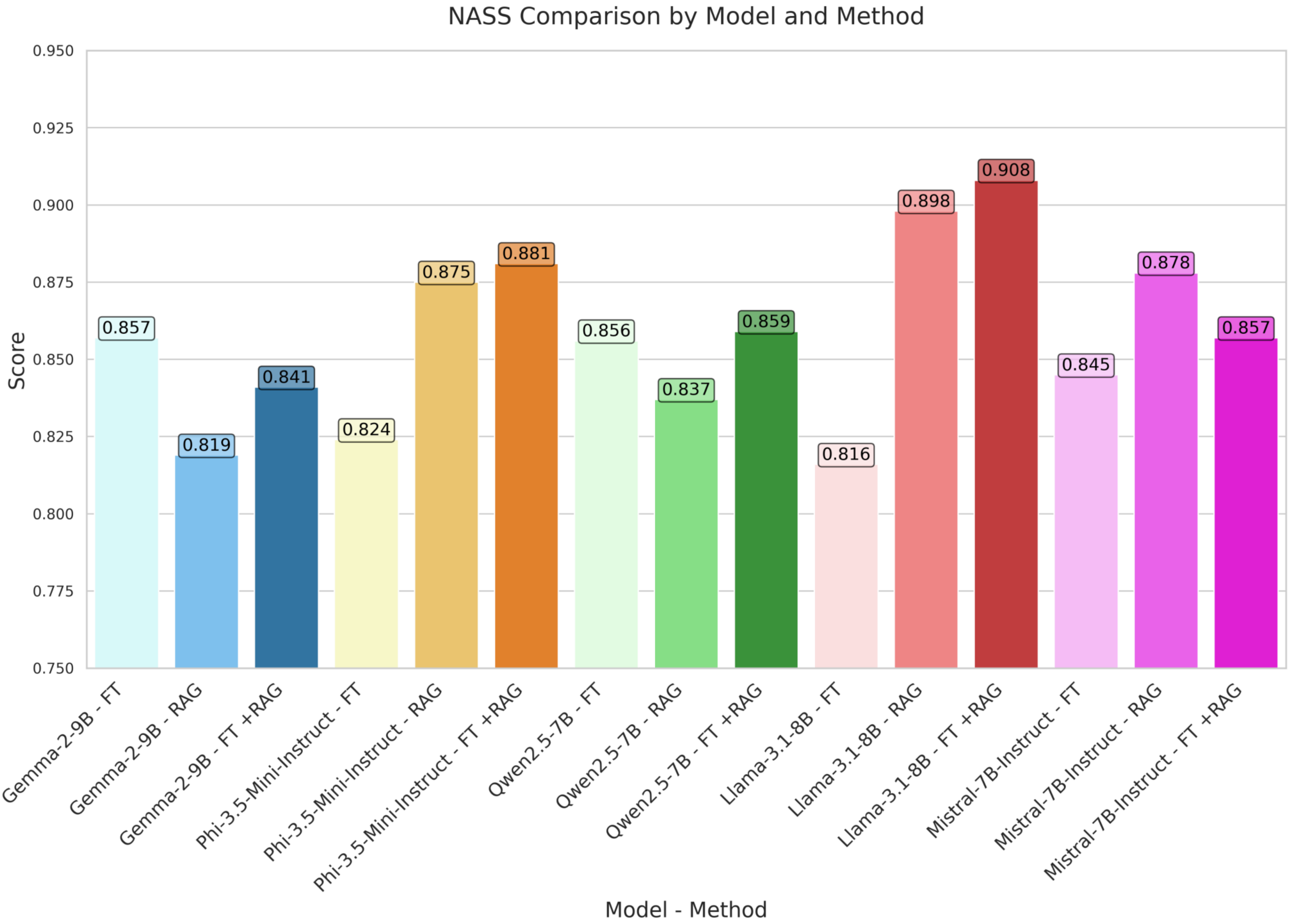

Figure S12: Model-wise Negation Aware Semantic Similarity Comparison: Fine-Tuning, RAG, and FT+RAG Strategies
